# CHMP4A in hepatocellular carcinoma: exploring its role in tumor progression, immune modulation, and potential link to TIM3 checkpoint

**DOI:** 10.3389/fimmu.2025.1682724

**Published:** 2025-10-02

**Authors:** Kai Sun, Song Wen, Shou-jun Guo, Qing-hua Pan, Ke-run Wang

**Affiliations:** Department of Oncology, Ganzhou Cancer Hospital, The Affiliated Cancer Hospital of Gannan Medical University, Ganzhou, Jiangxi, China

**Keywords:** CHMP4A, hepatocellular carcinoma, prognosis, immune infiltration, TIM3

## Abstract

**Background:**

Charged Multivesicular Body Protein 4A (CHMP4A), a member of the ESCRT-III family, plays a pivotal role in membrane remodeling and fission, with emerging evidence underscoring its significance in cancer immunotherapy. The complex pathogenesis and therapeutic resistance characteristic of liver hepatocellular carcinoma (LIHC) present significant challenges in clinical practice. This study investigated the potential involvement of CHMP4A in the progression of LIHC.

**Methods and results:**

Utilizing a comprehensive pan-cancer analysis with datasets from The Cancer Genome Atlas (TCGA), Gene Expression Omnibus (GEO), ArrayExpress database and the International Cancer Genome Consortium (ICGC), we evaluated the prognostic significance of CHMP4A, its clinical implications, associated signaling pathways, DNA methylation status, immune cell infiltration, and response to chemotherapy. Bioinformatics analyses, corroborated by immunohistochemical validation, revealed a marked overexpression of CHMP4A in LIHC specimens relative to adjacent normal tissues. Kaplan-Meier survival analyses indicated that this elevated expression pattern was associated with poor patient outcomes. Single-cell transcriptomic analysis had identified NK/T cells and tumor cells as the predominant cellular sources of CHMP4A within the LIHC microenvironment. Functional studies employing CHMP4A-specific small interfering RNA (siRNA) revealed significant inhibition of malignant phenotypes in LIHC cells, notably affecting proliferation, migration, and invasive capabilities. Mechanistically, the knockdown of CHMP4A led to modulation of the epithelial-mesenchymal transition (EMT), as indicated by the upregulation of E-cadherin and the concurrent downregulation of vimentin and matrix metalloproteinases (MMP-2/9). A comprehensive analysis of the immune landscape demonstrated significant correlations between CHMP4A expression patterns and various immunological parameters, including immune cell infiltration, expression of checkpoint molecules, tumor mutational burden (TMB), and microsatellite instability (MSI). Notably, the silencing of CHMP4A markedly decreased the expression levels of the TIM3/LGALS9 immune checkpoint axis in LIHC.

**Conclusions:**

Our extensive analyses identified CHMP4A as a critical molecular determinant in the progression of LIHC, which may function through two oncogenic mechanisms: the promotion of tumor cell proliferation and metastatic potential, and immunomodulatory effects associated with the TIM3/LGALS9 signaling pathway. These findings indicated that CHMP4A might serve as a potential therapeutic target and prognostic biomarker in LIHC.

## Introduction

Globally, LIHC is a major contributor to cancer-related mortality (1). Despite advances in medical research, the prognosis for LIHC patients remains poor, primarily due to limitations of existing therapies such as surgery, radiotherapy, and chemotherapy (2). These conventional treatments often fail to provide long-term relief and are accompanied by significant side effects. In recent years, immunotherapy has emerged as a promising approach to cancer treatment, harnessing the body’s immune system to target and destroy cancer cells (3). Among these approaches, immune checkpoint inhibitors (ICIs) targeting programmed death protein 1 (PD-1) and programmed death ligand 1 (PD-L1) have shown promising efficacy in liver cancer treatment (4–6). However, their application in liver cancer remains limited, with the main challenge being the liver’s unique immune microenvironment that may inhibit immunotherapeutic efficacy (4, 7). Additionally, response rates to immunotherapy remain low among liver cancer patients, with many exhibiting primary or secondary resistance to current immunotherapies (4). To enhance immunotherapy efficacy, researchers are exploring various strategies including combination therapies and novel immune targets. For instance, combining anti-PD-L1 antibodies with the vascular endothelial growth factor (VEGF) neutralizing antibody bevacizumab has become a first-line treatment option for LIHC (4). The immune microenvironment in liver cancer plays a crucial role in determining immunotherapy effectiveness. A deeper understanding of this microenvironment could facilitate more appropriate immunotherapy strategy selection (8).

The Endosomal Sorting Complex Required for Transport (ESCRT) machinery, with a particular emphasis on the ESCRT-III family, is integral to a variety of cellular functions, such as membrane remodeling, cytokinesis, and viral budding (9, 10). Within the realm of hepatic oncology, specifically HCC, the ESCRT-III complex has been associated with tumor progression and metastasis. Comprised of charged multivesicular body proteins (CHMPs), the ESCRT-III complex is vital for the formation of multivesicular bodies (MVBs) and the sorting of ubiquitinated proteins destined for degradation (11). This mechanism is critical for maintaining cellular homeostasis and averting oncogenic transformation. Recent research has underscored the critical role of CHMP family members in HCC. Specifically, CHMP3, a constituent of the ESCRT-III complex, has been implicated in the advancement of HCC by suppressing caspase-1-dependent pyroptosis, a type of programmed cell death. This suppression enables cancer cells to evade immune detection and continue proliferating, thereby facilitating tumor growth and metastasis (12). Additionally, the expression levels of CHMPs are markedly elevated in liver tumor tissues, which is associated with poor prognosis and increased drug resistance, indicating their potential as therapeutic targets (11).

CHMP4A is a member of the CHMP family members, which plays a crucial role in various cellular processes, including membrane remodeling and fission (9, 13, 14). Recent studies have highlighted its significance in cancer immunotherapy, particularly in breast cancer and lung cancer (15, 16). CHMP4A is a crucial gene linked to CD8+ T-cell infiltration in breast tumors and is associated with better survival rates in patients, indicating its potential as a prognostic marker. It enhances CD8+ T-cell recruitment by reducing LSD1 expression, leading to HERV dsRNA buildup and increased IFNβ and chemokine production. This process highlights CHMP4A’s role in boosting immune cell infiltration, thereby suppressing tumor growth and improving the effectiveness of immunotherapy in breast cancer (15). Beyond its role in cancer, CHMP4A, along with other ESCRT-III components, maintains the integrity of the endocytic network, which is critical for the envelopment of certain viruses, such as HSV1 (17). The ESCRT-III complexes, including CHMP4A, facilitates membrane scission, a process exploited by enveloped viruses during morphogenesis. This highlights the potential of targeting CHMP4A and related pathways as a therapeutic strategy against viral infections (17). Overall, CHMP4A’s dual involvement in immune modulation in cancer and membrane dynamics in viral infections positions it as a key regulator of cellular homeostasis and a promising therapeutic target. The clinical significance of CHMP4A expression in LIHC, especially its prognostic value, impact on immune cell infiltration, and therapeutic potential, is largely unexplored. This study aims to address this gap by examining CHMP4A’s role in LIHC progression and its interaction with the tumor immune microenvironment, establishing CHMP4A as a new biomarker and potential therapeutic target.

This study analyzed data from TCGA, GEO, and ICGC to explore CHMP4A expression and its prognostic value in LIHC. Immunohistochemistry showed higher CHMP4A levels in LIHC tissues compared to normal samples. DNA methylation, single-cell sequencing, and pharmacological profiling highlighted CHMP4A’s role in LIHC. *In vitro* assays demonstrated that CHMP4A knockdown reduced LIHC cell proliferation, invasion, and metastasis, affecting EMT markers like E-cadherin, vimentin, MMP-2, and MMP-9. The study also examined CHMP4A’s influence on the tumor immune microenvironment, linking it to TIM3 and LGALS9. Overall, the findings reveal CHMP4A’s crucial role in LIHC development and its therapeutic potential.

## Materials and methods

### Data collection, preprocessing, and expression analysis

Genomic datasets representing various cancer types were systematically obtained from TCGA repository (https://portal.gdc.cancer.gov/). To establish suitable control groups, normal tissue expression profiles were sourced from the Genotype-Tissue Expression (GTEx) database (http://gtexportal.org/) (18). Our analytical cohort included 110 normal liver specimens from GTEx, 50 paired non-tumorous adjacent tissues from hepatocellular carcinoma (HCC) patients, 371 HCC tumor samples, and their associated clinical metadata from TCGA-LIHC. Detailed clinicopathological characteristics of TCGA-LIHC are presented in **Supplementary Table S1**. To enhance the robustness of our findings, we integrated additional HCC datasets from the GEO platform (https://www.ncbi.nlm.nih.gov/geo/), specifically: GSE144269 (70 HCC tissues and 70 adjacent normal liver tissues), GSE14520 (22 HCC tissues and 22 paired non-tumor tissues, 42 HCC tissues and 22 paired non-tumor tissues), GSE54236 (81 HCC tissues and 80 non-tumor liver tissues), GSE76427 (115 HCC tissues and 52 normal liver tissues), GSE104580 (147 HCC tissues), GSE116174 (64 HCC tissues), and GSE109211 (140 HCC tissues) (19). The data from the ICGC were accessed via their official portal (https://dcc.icgc.org/). Additionally, the E_TABM_36 dataset, comprising 57 HCC samples, 3 adenomas, and 5 normal tissues, was obtained from ArrayExpress. Protein expression data for CHMP4A were sourced from the Human Protein Atlas (HPA, http://www.proteinatlas.org), which provides extensive immunohistochemical analyses across diverse tissue types (20, 21). All transcriptomic data were quantified in terms of transcripts per million (TPM) and subsequently normalized using a log2(TPM+1) transformation. All bioinformatics analyses were conducted in R software (version 4.3.0) with standardized workflows and parameters, ensuring reproducibility and alignment with established practices in cancer bioinformatics. Missing data were addressed using the missForest algorithm in R (22). Rigorous quality control measures were implemented, with potential outliers identified through interquartile range (IQR) analysis. Samples with values falling outside the thresholds of Q1 - 1.5×IQR or Q3 + 1.5×IQR were adjusted to the nearest valid data point (23). Only specimens with complete RNA-seq profiles and corresponding clinical information were included in the subsequent analyses. TCGA RNA-seq data (preprocessed as transcripts per million [TPM] values) were analyzed using the DESeq2 package (version 1.34.0) and the limma package (version 3.50.3) to account for RNA-seq-specific count distribution characteristics (24, 25). GEO microarray datasets were first normalized using the robust multi-array average (RMA) method to correct platform-specific biases. The statistical thresholds (adjusted *P*<0.05, |log_2_FC| > 1) were used to maintain consistency in DEG calling across datasets.

### Tissue samples and immunohistochemistry

A cohort of 16 matched pairs of LIHC specimens and corresponding adjacent non-tumor liver tissues was procured from Ganzhou Cancer Hospital. The study protocol received ethical clearance from the Institutional Review Board (Ethics Committee Approval No. 2025Kelunshen121). All cases were subjected to definitive histopathological confirmation for the diagnosis of LIHC. Detailed clinicopathological characteristics are presented in **Supplementary Table S2**. The inclusion criteria stipulated: (1) histologically confirmed LIHC, (2) availability of complete clinical records. Exclusion criteria included: (1) indeterminate pathological findings, (2) incomplete clinical data, (3) prior exposure to multiple lines of systemic therapy. For immunohistochemical analysis, tissue specimens were initially fixed in 10% neutral buffered formalin, embedded in paraffin blocks, and sectioned at a thickness of 4 μm. Following deparaffinization and rehydration, antigen retrieval was conducted using citrate buffer (1:100 dilution; Boster Biological Technology, China). Tissue sections were subsequently incubated with HRP-conjugated secondary antibodies (ZSGB-Bio, China), developed with 3,3′-diaminobenzidine (DAB) chromogen, and counterstained with hematoxylin. Quantitative image analysis was conducted using Image-Pro Plus 6.0 (Media Cybernetics, USA), with integrated optical density (IOD) measurements derived from multiple high-power fields per section.

### Prognosis analysis of CHMP4A

To evaluate patient outcomes, we conducted an extensive series of survival analyses using the Kaplan-Meier method, which facilitated the assessment of various clinical endpoints, including overall survival (OS), progression-free survival (PFS), disease-free survival (DFS), and disease-specific survival (DSS), between high- and low-CHMP4A expression groups (stratified by the median expression value of CHMP4A). These statistical analyses were performed utilizing the survival package (version 3.3-1). Log-rank tests were used to assess survival differences, with P < 0.05 considered statistically significant. The survminer package (version 0.4.9) was employed for survival curve visualization, including annotation of 95% confidence intervals (CIs) and median survival times for each group. The prognostic performance was further assessed through time-dependent ROC analysis using the “timeROC” package, which computed survival probabilities at 1-, 3-, and 5-year intervals, generated corresponding ROC curves, and determined their AUC values (26). To ensure the robustness of our findings concerning CHMP4A expression patterns in LIHC, we conducted external validation using independent cohorts from the GEO and ICGC repositories (19, 27). Additionally, we employed both univariate and multivariate Cox regression analyses to systematically evaluate potential prognostic indicators.

### Functional annotation analysis

To elucidate the biological significance of CHMP4A in LIHC, we conducted a comprehensive functional annotation utilizing Gene Ontology (GO) and KEGG pathway enrichment analyses (28). The GO analysis, a widely recognized framework in functional genomics, facilitated an extensive characterization of CHMP4A-associated biological processes, molecular functions, and subcellular localization patterns in LIHC (29). To gain deeper mechanistic insights, we employed Gene Set Enrichment Analysis (GSEA), a robust statistical method particularly adept at identifying coordinated expression changes in functionally related gene sets across different phenotypic states (30). All computational analyses were performed using advanced bioinformatics tools: the ClusterProfiler R package (version 3.14.3) was utilized for GO and KEGG pathway mapping, while GSEA software (version 4.1.0) was applied for pathway-level enrichment assessment. We employed the STRING database (STRING v9.1; http://string-db.org/newstring_cgi) to predict and systematically catalog protein-protein interactions (PPIs) among the concordant genes (31). Gene network analysis was performed using the GeneMANIA app within Cytoscape, which enables the import of interaction networks from public databases based on our candidate genes, along with their annotations and putative functions. The analytical pipeline adhered to established protocols to ensure reproducibility and statistical rigor in the interpretation of omics data. Results were filtered by FDR < 0.05 to minimize false positives.

### DNA methylation analysis

The EWAS Data Hub (https://ngdc.cncb.ac.cn/ewas/datahub/index) serves as a comprehensive database for epigenome-wide association studies, encompassing methylation profiles derived from 115,852 biological specimens across 528 distinct pathological conditions (32). In addition, the Shiny Methylation Analysis Resource Tool (SMART) platform (http://www.bioinfo-zs.com/smartapp/) offers an integrated analytical framework for processing data from the Infinium Human Methylation 450K array, transcriptomic sequencing, and clinical parameters across 33 malignancies documented in TCGA (33). These two bioinformatics resources were utilized to systematically assess CHMP4A epigenetic modifications in patients with hepatocellular carcinoma. Specifically, we examined the correlation between CHMP4A promoter methylation levels and its mRNA expression patterns, clinical characteristics, and prognostic implications for patient survival outcomes. Methylation data from Illumina HumanMethylation450K arrays were processed using the ChAMP package (version 2.22.0). Preprocessing included quality control: filtering probes with detection P-values > 0.01 (to exclude low-confidence signals), removing cross-reactive probes, and normalization via BMIQ (Beta Mixture Quantile Normalization). Methylation status was defined by beta-values (range: 0–1, reflecting methylated allele proportion): hypermethylation (beta > 0.6) and hypomethylation (beta < 0.2).

### Single-cell expression analysis

Single-cell transcriptomic profiling was conducted using transcriptomic data in.h5 format, which included detailed cellular annotations obtained from the TISCH repository (34). The subsequent computational analyses were performed using the MAESTRO framework and the Seurat toolkit within the R statistical environment to ensure comprehensive data preprocessing and quality assessment. Cellular dimensionality reduction and population stratification were accomplished through the application of the t-SNE computational approach. For the analysis of the GSE149614 cohort, RNA sequencing data from 10 HCC samples were subjected to standardized processing protocols. These protocols included normalization procedures, variable feature identification, and unsupervised classification to delineate heterogeneous cell populations. The computational methodology employed rigorous quality control parameters to ensure data integrity throughout all stages of analysis. The Seurat package (version 4.1.0) handled preprocessing and clustering. Preprocessing included filtering low-quality cells (excluding those with <200 detected genes, >5% mitochondrial gene content, or >2000 detected genes to remove doublets/apoptotic cells), normalization via LogNormalize, and scaling. Cells were clustered using the Louvain algorithm (resolution = 0.5, optimized for granularity and biological relevance) and annotated via canonical markers (e.g., AFP for hepatocytes, CD3D for T cells). CHMP4A expression across clusters was visualized using Uniform Manifold Approximation and Projection (UMAP) and violin plots.

### Immune correlation analysis

To investigate the relationship between CHMP4A and tumor microenvironment characteristics in LIHC, we performed an extensive analysis of immune infiltration using datasets from the TCGA, GEO, ICGC and ArrayExpress repositories. Our computational approach integrated several R packages, including “GSVA,” “immunedeconv,” “estimate,” “ggplot2,” “pheatmap,” and “ggstatsplot,” to evaluate patterns of immune cell infiltration, stromal components, immune activity scores, and genomic instability markers such as TMB and MSI. The study utilized eight advanced computational techniques: ssGSEA, xCell, CIBERSORT, EPIC, TIMER, MCP-counter, and quanTIseq. Additionally, we conducted a systematic analysis of the correlation between CHMP4A expression and 150 well-characterized immune-related genes across five key immunological pathways: chemokine signaling (41 genes), immune receptor activity (18 genes), MHC complex (21 genes), immunosuppressive factors (24 genes), and immunostimulatory molecules (46 genes) (35–37). All statistical analyses and visualizations were executed using R statistical software (version 4.3.0).

### Drug sensitivity of CHMP4A in LIHC

Extensive pharmacological sensitivity data were obtained from three reputable public repositories: the Cancer Therapeutics Response Portal (CTRP v2.0) (https://portals.broadinstitute.org/ctrp.v2.1/), the PRISM Repurposing dataset (https://www.theprismlab.org/), and the Genomics of Drug Sensitivity in Cancer (GDSC) database (https://www.cancerrxgene.org/). To systematically explore the relationship between CHMP4A expression levels and therapeutic response, we utilized Spearman’s correlation analysis to evaluate potential associations with 217 pharmacological agents, including kinase inhibitors, epigenetic modulators, and conventional chemotherapeutic drugs. All bioinformatics analyses were conducted using R statistical software (version 4.3.0), employing the tidyverse ecosystem for data processing, the pRRophetic algorithm for drug response modeling, and ComplexHeatmap for detailed visualization of the results (38).

### Cell culture

Human hepatocellular carcinoma HEP3B2.1–7 cell line were purchased from Sangon Biotech (Shanghai, China). HEP 3B2.1–7 cells were cultured in grown in MEM (Procell, PM150410) supplemented with 10% fetal bovine serum (FBS, Gibco, USA), with additional 1% penicillin/streptomycin (Solarbio, China) at 37°C humidified incubator containing 5% CO^2^.

### siRNA transfection for CHMP4A knockdown

Twenty-four hours prior to transfection, HEP 3B2.1–7 cells were seeded into 6-well culture plates and cultured until reaching 80-90% confluency. Transfection was conducted using Lipofectamine reagent (KeyGEN, China) in accordance with the manufacturer’s protocol, employing either target-specific siRNA or a negative control (NC). Cellular RNA and protein were extracted 24 hours post-transfection for subsequent analyses. The experimental design comprised six distinct treatment conditions: untreated HEP 3B2.1–7 cells (CTRL group), negative control siRNA (siNC), four different CHMP4A-targeting siRNAs si-CHMP4A-1 (siCHMP4A-214), si-CHMP4A-2 (siCHMP4A-316), si-CHMP4A-3 (siCHMP4A-416), and si-CHMP4A-1 (siCHMP4A-563). Following preliminary screening, siCHMP4A-316 (designated as si-CHMP4A-2) exhibited superior knockdown efficiency and was thus selected for further functional characterization studies.

The siRNA sense used were as follows:

siCHMP4A-214: sense:5’-GACCAAGAAUAAGAGAGCUTT-3’,
antisense: 5’-AGCUCUCUUAUUCUUGGUCTT-3’.
siCHMP4A-316: sense:5’-GCGUGAGGCCAUUGAGAAUTT-3’,
antisense:5’-AUUCUCAAUGGCCUCACGCTT-3’.
siCHMP4A-416: sense:5’-GACAAGGUAGAUGAACUGATT-3’,
antisense:5’-UCAGUUCAUCUACCUUGUCTT-3’.
siCHMP4A-563: sense:5’-GAAUUGGCCCAGGAGUUGUTT-3’,
antisense:5’-ACAACUCCUGGGCCAAUUCTT-3’.
The sense of negative control RNA (NC) was as follows:
siNC: sense:5’-UUCUCCGAACGUGUCACGUTT-3’,
antisense:5’-ACGUGACACGUUCGGAGAATT -3’.

### Real-time quantitative PCR assay

Total RNA was extracted utilizing the RNA Isolater Total RNA Extraction Reagent (VAZYME) in accordance with the manufacturer’s instructions. Subsequently, the isolated RNA was reverse transcribed into complementary DNA (cDNA) using the HiScript^®^ II Q RT SuperMix for qPCR (+gDNA wiper) (VAZYME). Quantitative PCR (qPCR) analysis was conducted on the synthesized cDNA samples employing the ChamQ SYBR qPCR Master Mix (VAZYME). Gene expression levels were quantified by determining relative expression through the comparative CT (2^-ΔΔCT) method. The primer sequences used were as follows:

CHMP4A: forward, 5’- TACAGGCTTTGCGGAGGAAG -3’;
reverse, 5’- CACCTCCTGTTGTTCCGTGA -3’, 227bp.
GAPDH: forward, 5’-ATGGGGAAGGTGAAGGTCGGAGT-3’;
reverse, 5’- TAGTTGAGGTCAATGAAGGGGTC-3’, 125bp.

### Western blot analysis for protein expression detection

After transfection, cellular samples underwent two washes with phosphate-buffered saline (PBS) and were subsequently lysed using ice-cold RIPA buffer. Protein quantification was conducted utilizing a BCA protein detection kit (GBCBIO, China). Proteins were then separated via electrophoresis on 10% SDS-polyacrylamide gels and transferred to nitrocellulose membranes (Biofroxx, Germany). Membrane blocking was performed with 5% non-fat milk for 2 hours at room temperature, followed by an overnight incubation at 4°C with specific primary antibodies. Between incubations, the membranes were washed three times for 10 minutes each with Tris-buffered saline containing Tween-20 (TBST). Secondary detection was carried out using HRP-conjugated goat anti-mouse IgG (1:10000 dilution, Boster, China), with the same washing conditions applied prior to chemiluminescent development. The immunoblotting analysis included the following primary antibodies: anti-rabbit CHMP4A (1:1000, SinoBiological, China), anti-mouse GAPDH (1:30000, Proteintech, China), anti-rabbit E-cadherin (1:40000, Proteintech, China), anti-mouse Vimentin (1:40000, Proteintech, China), anti-rabbit MMP2 (1:1000, BIOSS, China), anti-rabbit MMP9 (1:1000, Affinity, China), anti-rabbit TIM3(HAVCR2) (1:1000, Boster, China) and anti-rabbit LGALS9 (1:1000, Abmat, China).

### Assessment of cell proliferation

The kinetics of cellular growth were assessed utilizing the CCK-8 assay system (HYCEZMBIO, China). Post-transfection, cells were plated into 96-well culture plates at an initial density of 3×10³ cells per well. At specified intervals (0, 24, 36 and 48 hours post-seeding), 10 μL of CCK-8 reagent was introduced to each well, followed by incubation under standard culture conditions (37°C, 5% CO_2_) for 60 minutes. Absorbance was subsequently measured at a wavelength of 450 nm using a microplate spectrophotometer (Thermo Scientific, USA).

### Transwell assays for cell migration and invasion

For the migration and invasion assay, a 24-well Transwell system (Corning, USA) equipped with 8 µm porous membranes pre-coated with 100 μL of Matrigel basement membrane matrix (Corning, USA) was employed. The experimental protocol involved seeding 6×10^4 transfected HEP 3B2.1–7 cells, suspended in a serum-free medium, into the upper chamber, while the lower chamber was filled with 600 μL of complete medium containing 20% fetal bovine serum to serve as a chemoattractant. Following a 24-hour incubation period at 37°C, cells that had successfully traversed the membrane were fixed using 4% paraformaldehyde for 1 hour and subsequently stained with a 0.5% crystal violet solution for 20 minutes. Quantitative analysis was conducted by counting the stained cells in five randomly selected microscopic fields per membrane using bright-field microscopy.

### Statistical analysis methods

Statistical analyses were conducted using the R statistical software (version 4.3.0), incorporating a variety of methodological approaches to ensure a thorough evaluation of the data. Quantitative analyses involved the calculation of fold-change values and hazard ratios (HR), supplemented by significance probabilities derived from Log-rank tests. Bivariate relationships were assessed using both Spearman’s rank-order and Pearson’s product-moment correlation coefficients. Comparative analyses between experimental groups were performed using Wilcoxon rank-sum tests, Student’s t-tests for pairwise comparisons, and analysis of variance for multi-group comparisons. Time-to-event data were depicted using Kaplan-Meier survival curves, accompanied by log-rank test statistics, with a conventional significance threshold set at *P*=0.05. Graphical annotations employed an asterisk-based system to denote statistical significance: * (*P*<0.05), ** (*P*<0.01), *** (*P*<0.001), and **** (*P*<0.0001).

## Results

### Assessment of CHMP4A expression and its link to clinical parameters in LIHC using public databases and experimental validation

**Figure 1:**
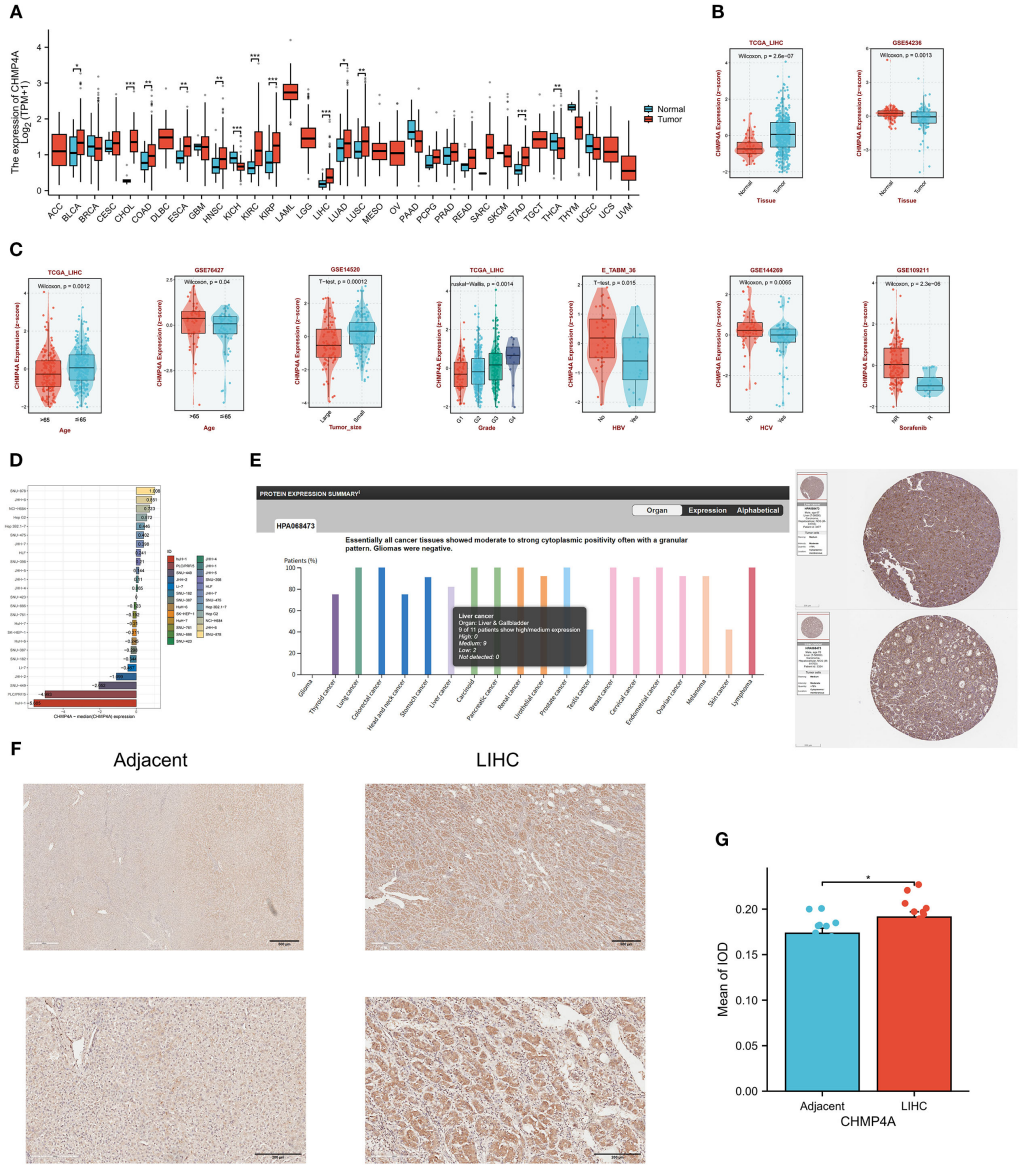
Expression Analysis of CHMP4A and Its Association with Clinical Features **(A)** Comparative expression analysis of CHMP4A in pan-cancer tissues versus adjacent normal tissues utilizing data from the TCGA and GETx databases. **(B)** Comparative expression analysis of CHMP4A in tumor versus normal tissues in LIHC using data from the TCGA and GEO databases. **(C)** Examination of the association between CHMP4A expression and clinical parameters in LIHC. **(D)** Comparative expression analysis of CHMP4A in hepatocellular carcinoma cell lines. **(E)** Pan-cancer protein expression profiling of CHMP4A, including representative IHC staining of tissue microarrays from the HPA database. **(F)** IHC analysis of CHMP4A in LIHC tumor tissues compared to paired adjacent non-tumor liver tissues. **(G)** Quantification of CHMP4A immunostaining using integrated optical density (IOD) analysis. *P<0.05, **P<0.01, ***P<0.001. CHMP4A, Charged Multivesicular Body Protein 4A; LIHC, liver hepatocellular carcinoma; TCGA, The Cancer Genome Atlas; GEO, Gene Expression Omnibus; AFP, Alpha-fetoprotein; IHC, immunohistochemistry; HPA, Human Protein Atlas; IOD, integrated optical density.

The study design flowchart is depicted in **Table 1**. An analysis of CHMP4A transcript levels across 33 cancer types, utilizing data from the TCGA database with corresponding normal tissue data from the GTEx database, revealed significant heterogeneity between tumors and adjacent normal tissues. The analysis indicated that CHMP4A mRNA expression was significantly elevated in several cancer tissues compared to normal tissues (**Figure 1A**), notably in bladder urothelial carcinoma (BLCA), cholangiocarcinoma (CHOL), colon adenocarcinoma (COAD), esophageal carcinoma (ESCA), head and neck squamous cell carcinoma (HNSC), kidney papillary cell carcinoma (KIRP), kidney renal clear cell carcinoma (KIRC), liver hepatocellular carcinoma (LIHC), lung adenocarcinoma (LUAD), lung squamous cell carcinoma (LUSC), and stomach adenocarcinoma (STAD). Conversely, CHMP4A expression was found to be lower in cancer tissues compared to corresponding normal tissues only in kidney chromophobe (KICH) and thyroid carcinoma (THCA). The expression of CHMP4A was upregulated across a wide range of tumor types; however, it was notably downregulated in KICH and THCA. This unique expression profile highlighted the context-dependent functional roles of CHMP4A in various cancer types, providing a foundation for further research. Future studies were suggested to aim to elucidate the potential tumor-suppressive role of CHMP4A in KICH and THCA, which might reveal cancer-type-specific regulatory mechanisms. We subsequently examined the expression patterns of CHMP4A in LIHC and their clinical correlations across multiple datasets (**Figures 1B, C**). Analysis of TCGA-LIHC samples revealed significantly elevated CHMP4A expression in tumor tissues compared to adjacent normal tissues (*P*=2.6e-07); however, this trend was reversed in the GSE54236 dataset (**Figure 1B**). Variations in CHMP4A expression across databases such as TCGA and GEO might have resulted from cohort-specific differences, including sample size, tumor stage, etiological factors (e.g., HBV/HCV status), and technical differences in RNA sequencing methods and data normalization. To address these inconsistencies and validate our findings, we used additional datasets such as the HPA database and conducted immunohistochemical staining on clinical LIHC samples to confirm CHMP4A expression in LIHC. Notably, age-related expression patterns exhibited cohort-specific variations: patients over 65 years of age showed increased CHMP4A levels in the GSE76427 dataset, whereas a decrease in expression was observed in the TCGA-LIHC cohort (**Figure 1C**). A consistent correlation was found between tumor grade and CHMP4A expression in TCGA-LIHC, with higher-grade tumors (G3/G4) demonstrating elevated expression relative to lower-grade tumors (G1/G2) (**Figure 1C**). Furthermore, analysis of tumor size in the GSE14520 dataset indicated an inverse relationship, whereby larger tumors were associated with reduced CHMP4A expression. The status of viral hepatitis infection also affected expression levels; patients positive for HBV/HCV exhibited lower CHMP4A expression in the E_TABM_36 and GSE144269 datasets compared to non-infected individuals. Additionally, treatment response data from the GSE109211 dataset revealed that Sorafenib non-responders had higher CHMP4A expression compared to responders. The analysis of hepatocellular carcinoma cell lines demonstrated significant variability in expression levels, with SNU-449, JHH-2, NCI-H684, Hep G2, and Hep 3B2.1–7 exhibiting relatively high expression, whereas Huh-1 and PLC/PRF/5 showed lower expression levels (**Figure 1D**).

**Table 1:** The research flowchart of this study.

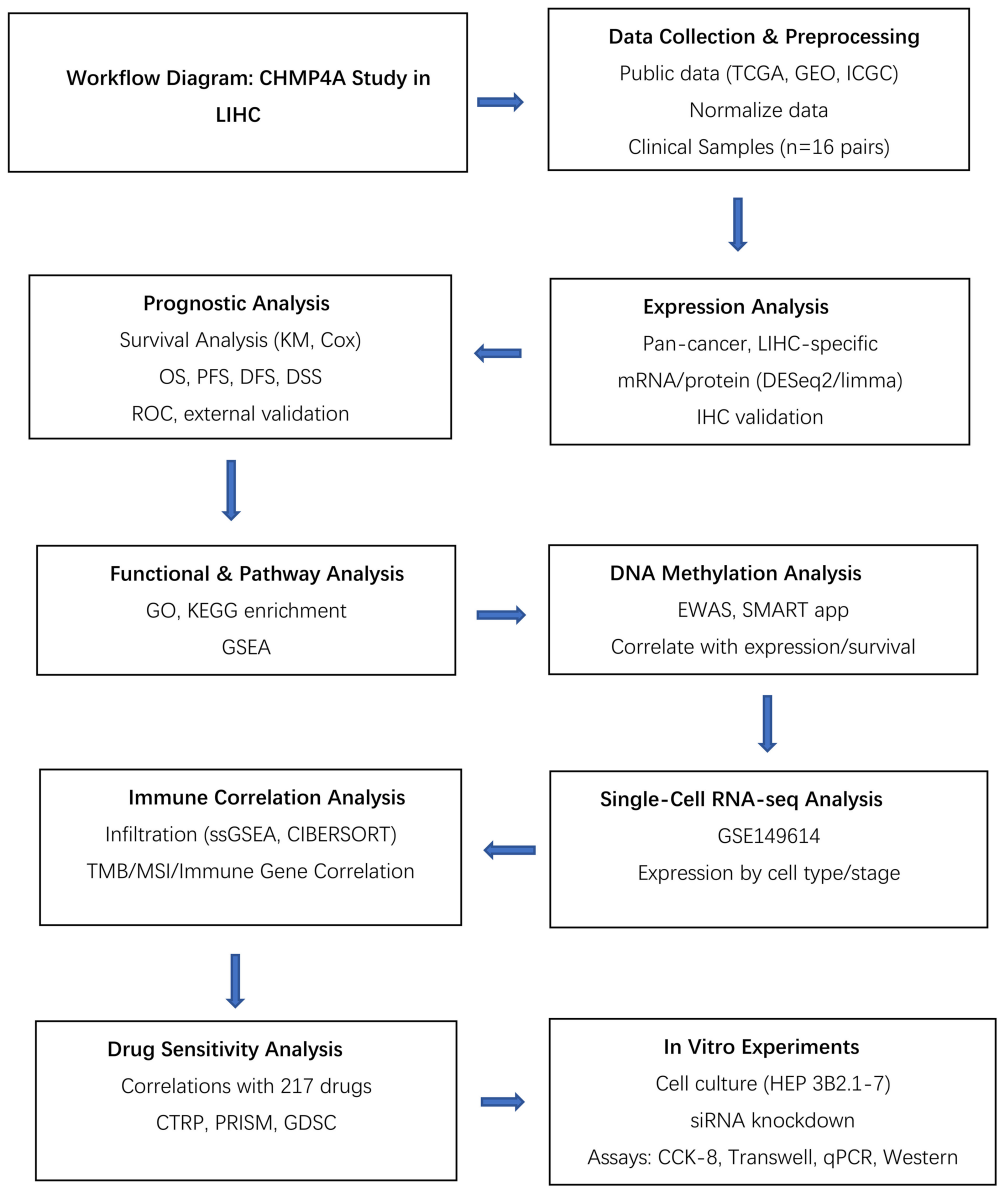	

To validate the mRNA findings at the protein level, an analysis of data from the HPA database was conducted, which demonstrated moderate to strong cytoplasmic positivity with a granular pattern in nearly all cancer tissues (**Figure 1E**). Notably, patients with LIHC exhibited significant immunoreactivity, with moderate positivity detected in two cases (**Figure 1E**). *In vitro* experiments, IHC analysis of 16 paired LIHC and adjacent normal tissue samples revealed that CHMP4A proteins were primarily localized in the cytoplasm of LIHC cells, as indicated by the presence of brown staining. In contrast, the expression of CHMP4A was notably weaker in the normal tissues. (**Figure 1F**). Quantification of CHMP4A protein levels, assessed through integrated optical density (IOD) values, confirmed a significantly higher expression in LIHC tissues compared to adjacent non-tumor tissues (*P*<0.05) (**Figure 1G**).

### Prognostic significance of CHMP4A expression across pan-cancer, with emphasis on LIHC

**Figure 2:**
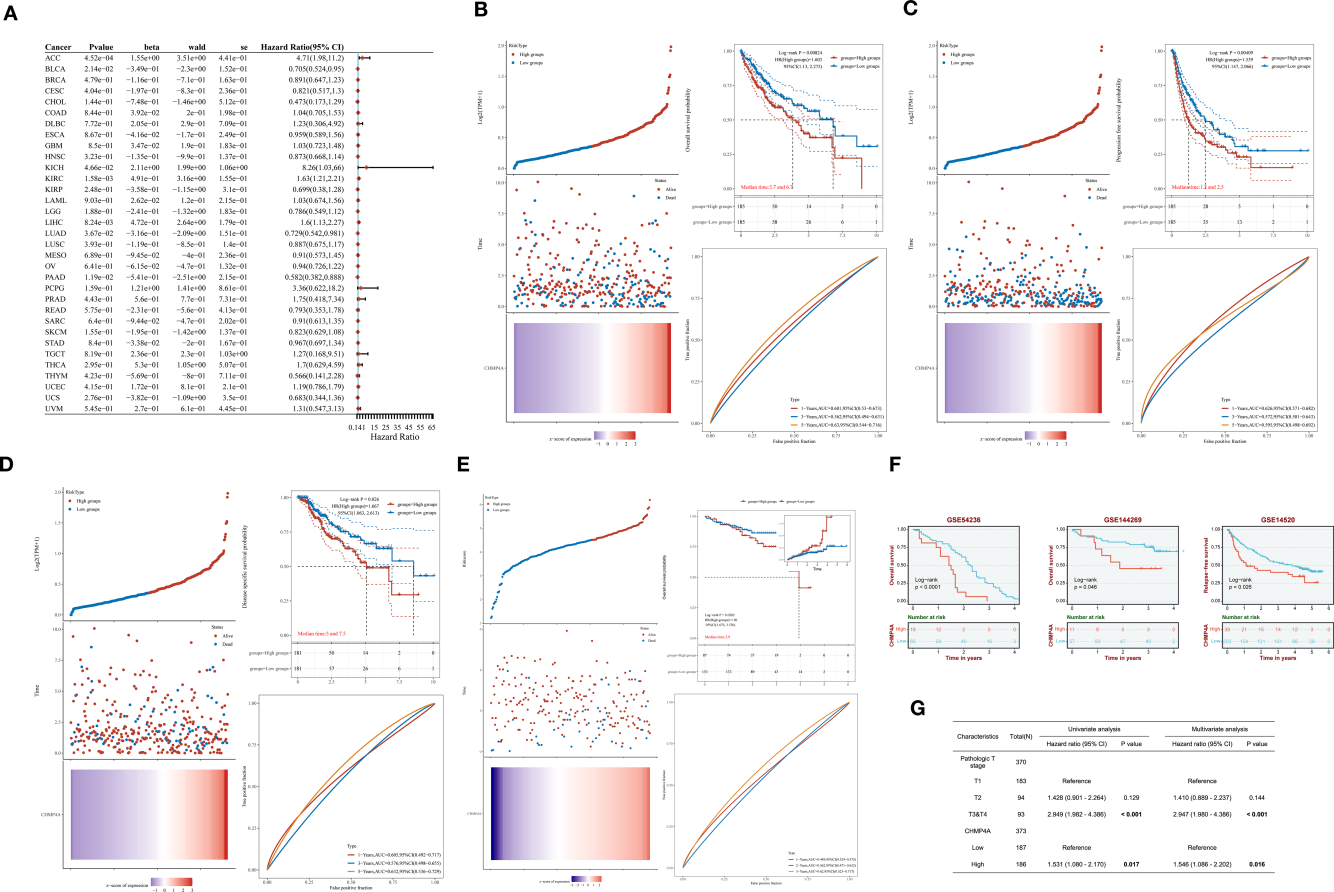
The prognostic relevance of CHMP4A expression was investigated across various cancers, with a specific focus on validation within LIHC cohorts. **(A)** A comprehensive pan-cancer Cox regression analysis was conducted to evaluate CHMP4A expression. **(B)** OS analysis of CHMP4A was performed using TCGA-LIHC data. **(C)** PFS analysis of CHMP4A was conducted utilizing TCGA-LIHC data. **(D)** DSS analysis of CHMP4A was carried out with TCGA-LIHC data. **(E)** OS analysis of CHMP4A was also performed using data from the ICGC. **(F)** External validation using independent GEO cohorts confirmed the prognostic significance of CHMP4A in LIHC. **(G)** The prognostic significance of CHMP4A expression in LIHC patients was further assessed through both univariate and multivariate analyses. AUC, Area Under Curve; CI, Confidence Interval; DFS, Disease-Free Survival; GEO, Gene Expression Omnibus; HR, Hazard Ratio; LIHC, Liver Hepatocellular Carcinoma; OS, Overall Survival; PFS, Progression-Free Survival; RFS, Relapse-Free Survival; ROC, Receiver Operating Characteristic; TCGA, The Cancer Genome Atlas; TPM, Transcripts Per Million; ICGC, International Cancer Genome Consortium.

Upon identifying the aberrant expression of CHMP4A in LIHC, we undertook an investigation into its prognostic significance. Our comprehensive pan-cancer univariate Cox regression analysis, encompassing 33 cancer types, demonstrated that elevated CHMP4A expression was significantly associated with poorer overall survival (OS) in several cancers, including adrenocortical carcinoma (ACC; hazard ratio [HR] = 4.71, *P*=4.52e−04), KICH (HR=8.26, *P* =4.66e−02), KIRC (HR=1.63, *P*=1.58e−03), and LIHC (HR=1.6, *P*=8.24e−03). Conversely, an inverse relationship was observed in BLCA (HR=0.705, *P*=2.14e−02), LUAD (HR=0.729, P=3.67e−02), and pancreatic adenocarcinoma (PAAD; HR=1.85, P=1.19e−02) (**Figure 2A**). Specifically focusing on LIHC, patients exhibiting high CHMP4A expression consistently experienced poorer clinical outcomes across various survival metrics, including OS (HR=1.603, *P*=0.00824), progression-free survival (PFS; HR=1.539, *P*=0.00409), and disease-specific survival (DSS; HR=1.667, *P*=0.026) in TGCA-LIHC (**Figures 2B–D**). This association was corroborated by data from the ICGC database, where elevated CHMP4A expression similarly predicted worse OS (HR=1.96, *P*=0.0282) (**Figure 2E**). Validation studies utilizing three independent GEO datasets (GSE54236, GSE144269, GSE14520) consistently demonstrated that patients exhibiting high CHMP4A expression experienced significantly reduced median OS compared to those in low-expression groups (*P*<0.05) (**Figure 2F**). Univariate analysis revealed that high CHMP4A expression significantly increased the risk for OS (HR=1.531, *P*=0.017), a finding confirmed by multivariate analysis (HR=1.546, *P*=0.016). Additionally, pathologic T stage (T3/T4 vs. T1/T2) was an independent prognostic factor (*P*<0.001) (**Figure 2G**).

### DNA methylation analysis of CHMP4A in LIHC patients

**Figure 3:**
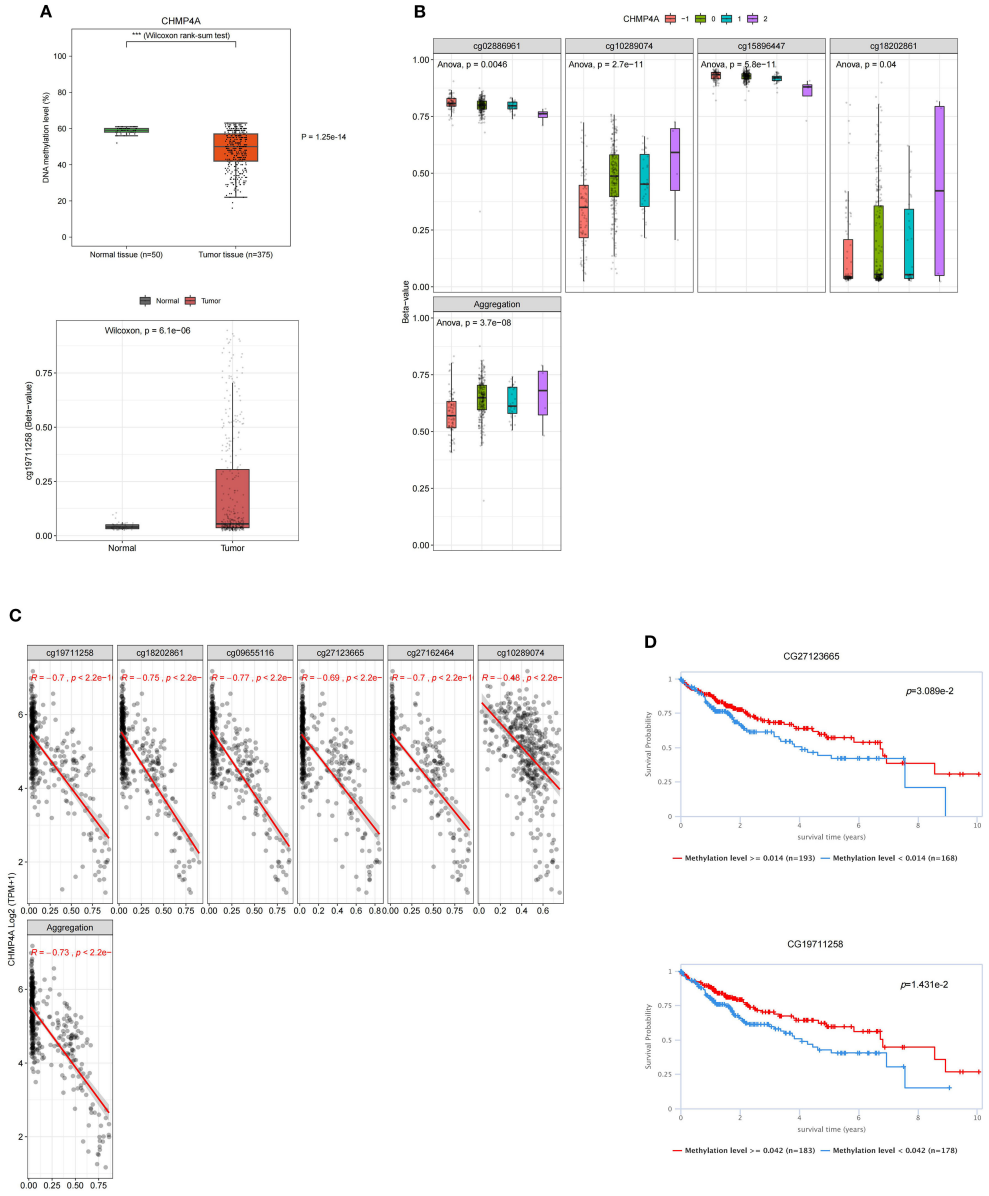
DNA methylation profiling of CHMP4A genomic features in LIHC. **(A)** Dynamics of promoter methylation in LIHC versus normal liver tissues. **(B)** Correlation between methylation levels of individual CHMP4A CpG sites and CNV status [deep deletion (-2), loss (-1), neutral (0), gain (1), amplification (2)]. **(C)** Correlation analysis between CHMP4A expression and its methylation status. **(D)** Association of CHMP4A methylation with patient survival outcomes. ***P<0.001. CpG, Cytosine-phosphate-Guanine dinucleotide; LIHC, Liver Hepatocellular Carcinoma; TCGA, The Cancer Genome Atlas; TNM, Tumor-Node-Metastasis staging system; CNV, copy number variation.

DNA methylation plays a crucial role in mediating the phenotypic alterations and clinical manifestations observed in LIHC, exerting profound impacts on tumor biology and patient outcomes. Building on the observed aberrant CHMP4A expression, we next examined its epigenetic regulation through DNA methylation analysis to elucidate potential mechanisms underlying its overexpression in LIHC. By systematically analyzing CHMP4A gene methylation patterns using the EWAS Data Hub and SMART APP platforms, we identified strong associations between epigenetic modifications, transcriptional regulation, and clinical parameters in LIHC patients. The genomic structure of CHMP4A revealed ten functionally significant CpG methylation sites within CpG island domains (**Supplementary Figures S1A, B**). Comparative analyses indicated significant lower methylation levels (hypomethylation) of the CHMP4A promoter region in malignant tissues compared to normal tissues (**Figure 3A**), whereas the cg19711258 site exhibited an opposite methylation pattern. While overall promoter methylation is lower in tumors, specific sites like cg19711258 may become hypermethylated, blocking key transcription activators and silencing CHMP4A expression. Further investigations into this aspect will be conducted in our subsequent research. An integrative analysis of copy number variation (CNV) and methylation profiles revealed significant interactions between epigenetic and genomic factors. Different CNV states (deletion, neutral, gain, amplification) were associated with distinct methylation patterns, with CpG sites cg02886961, cg10289074, cg15896447, and cg18202861 demonstrating CNV-dependent methylation variations (**Figure 3B**). Global methylation patterns further substantiated this robust association, indicating a coordinated genomic and epigenetic dysregulation in LIHC tumorigenesis. Transcriptional analysis identified six CpG sites (cg19711258, cg18202861, cg09655116, cg27123665, cg27162464, cg10289074) that exhibited significant inverse correlations between methylation density and CHMP4A expression levels (**Figure 3C**). The prognostic significance of CHMP4A methylation was evaluated by presenting Kaplan-Meier survival curves for two CpG sites, CG27123665 and CG19711258. Patients exhibiting elevated methylation levels at these sites demonstrated significantly improved survival outcomes, highlighting the potential utility of CHMP4A methylation as a prognostic biomarker for LIHC (**Figure 3D**). These findings collectively identified CHMP4A methylation as a molecular determinant in the progression of LIHC, thereby providing potential diagnostic and prognostic value for clinical management.

### Enrichment analysis of genes co-expressed with CHMP4A in LIHC

**Figure 4:**
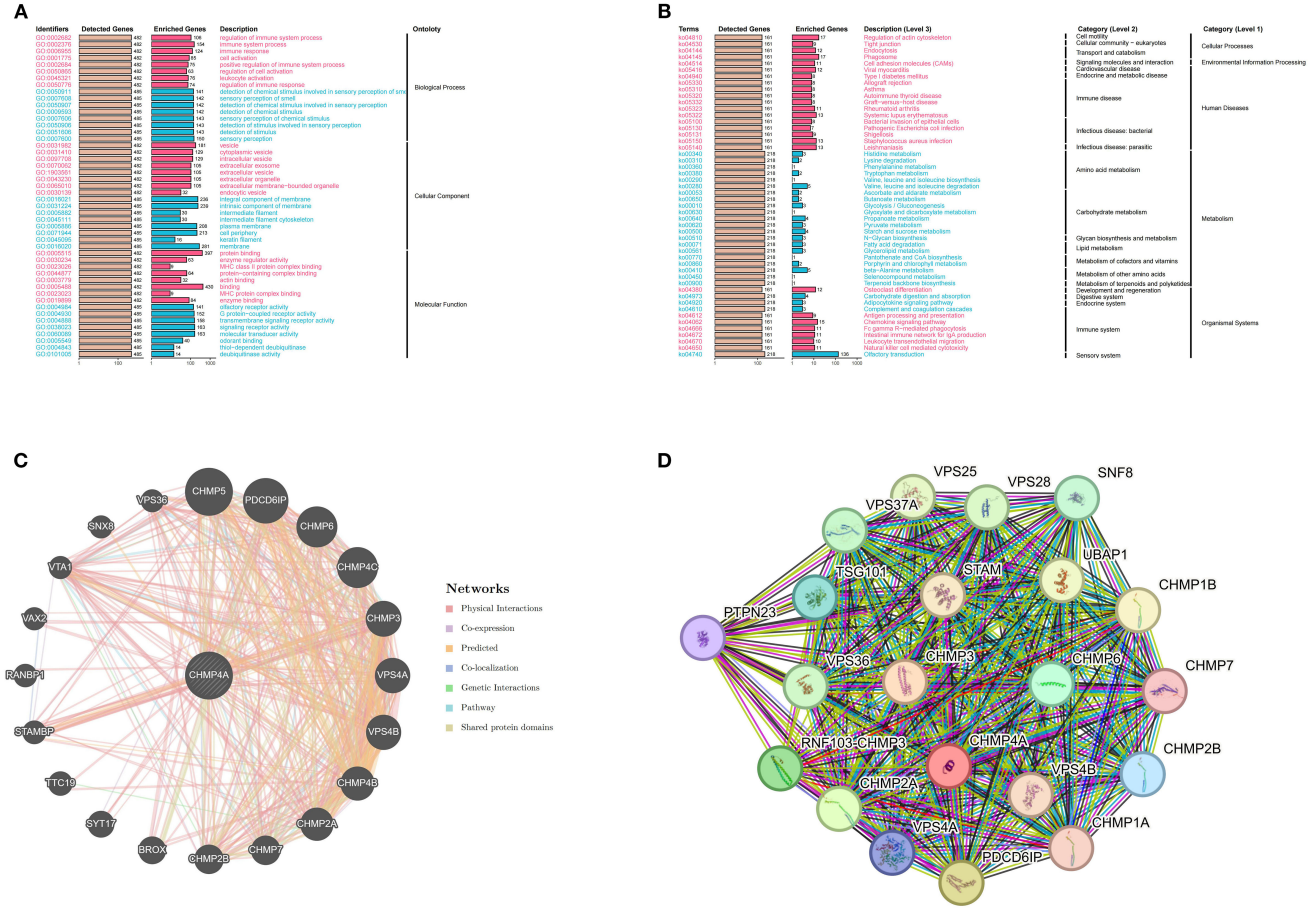
CHMP4A functional enrichment analysis across immune-related pathways and biological processes in LIHC. **(A)** GO enrichment analysis of CHMP4A associated biological processes. **(B)** KEGG pathway enrichment of CHMP4A. **(C)** Protein-protein interaction network analysis of CHMP4A in LIHC. **(D)** Gene Co-Expression Network Correlated with CHMP4A Expression Patterns in LIHC. LIHC, Liver Hepatocellular Carcinoma; CHMP4A, Charged Multivesicular Body Protein 4A; ES, Enrichment Score; GSEA, Gene Set Enrichment Analysis; GO, Gene Ontology; KEGG, Kyoto Encyclopedia of Genes and Genomes; CHMP4A, Charged Multivesicular Body Protein 4A; TCGA, The Cancer Genome Atlas.

To elucidate the biological function of CHMP4A in LIHC, we performed a comprehensive GSEA utilizing GO terms, KEGG pathways, and Hallmark gene sets. The findings revealed distinct patterns of pathway activation associated with different CHMP4A expression levels (**Figure 4**). The GO analysis identified statistically significant associations across various biological categories (**Figure 4A**). Notably, there was a significant overrepresentation of immune-related processes, such as “modulation of immune system activity”, “immune system process” and “immune response” suggesting a potential regulatory role for CHMP4A in immune mechanisms. Analysis of cellular components (CC) indicated enrichment in “extracellular vesicles” and “intrinsic membrane components” suggesting involvement in intercellular signaling and membrane-related activities. Furthermore, the molecular function (MF) analysis revealed enrichment for “macromolecular binding” and “catalytic activity regulation” highlighting the protein’s diverse functional capabilities. The evaluation of KEGG pathways revealed significant enrichment in critical signaling pathways, including “Actin cytoskeleton modulation”, “Vesicular phagocytosis” and “Chemotactic cytokine signaling” indicating the involvement of CHMP4A in cellular trafficking and immune responses (**Figure 4B**). Quantitative GSEA metrics demonstrated upregulation of pathways such as “leukocyte adhesion enhancement”, “cellular activation stimulation” and “RNA splicing machinery”, while pathways related to “branched-chain amino acid degradation” and “eicosanoid metabolism” were downregulated (**Supplementary Figures S2A, B**), further corroborating these findings. These results suggested that CHMP4A may play a dual role in facilitating immune cell communication and regulating metabolic homeostasis. Hallmark signature analysis confirmed significant enrichment in immunological pathways (“Transplant rejection”, “Cell plasticity transition” and “Interferon-γ signaling”) as well as fundamental cellular processes (“Programmed cell death”, “Genomic stability maintenance” and “Cell cycle regulation”), underscoring the multifaceted functions of CHMP4A in the pathogenesis of LIHC (**Supplementary Figure S2C**). Utilizing integrative analysis with the STRING and GeneMANIA databases, we systematically mapped the functional interactome of CHMP4A to elucidate its molecular characteristics (**Figures 4C, D**). The resulting protein-protein interaction networks revealed that CHMP4A forms robust associations with numerous molecular partners. Notably, key interactors such as VPS36, CHMP5, and PDCD6IP were identified, all of which are integral to vesicular transport mechanisms and immune-related signaling pathways (**Figure 4C**). Functional clustering analysis via GeneMANIA categorized these molecular associations into distinct biological modules. The findings underscored the significant involvement of VPS20, VPS28, and several CHMP family proteins (including CHMP1A, CHMP1B, and CHMP7), suggesting that CHMP4A likely mediates its biological effects in LIHC through complex cooperative interactions with these molecular partners (**Figure 4D**). These extensive interaction networks may collectively regulate fundamental cellular activities such as intracellular vesicle transport and membrane dynamics—processes frequently disrupted in malignant conditions.

### Single-cell RNA sequencing analysis of CHMP4A expression in LIHC

**Figure 5:**
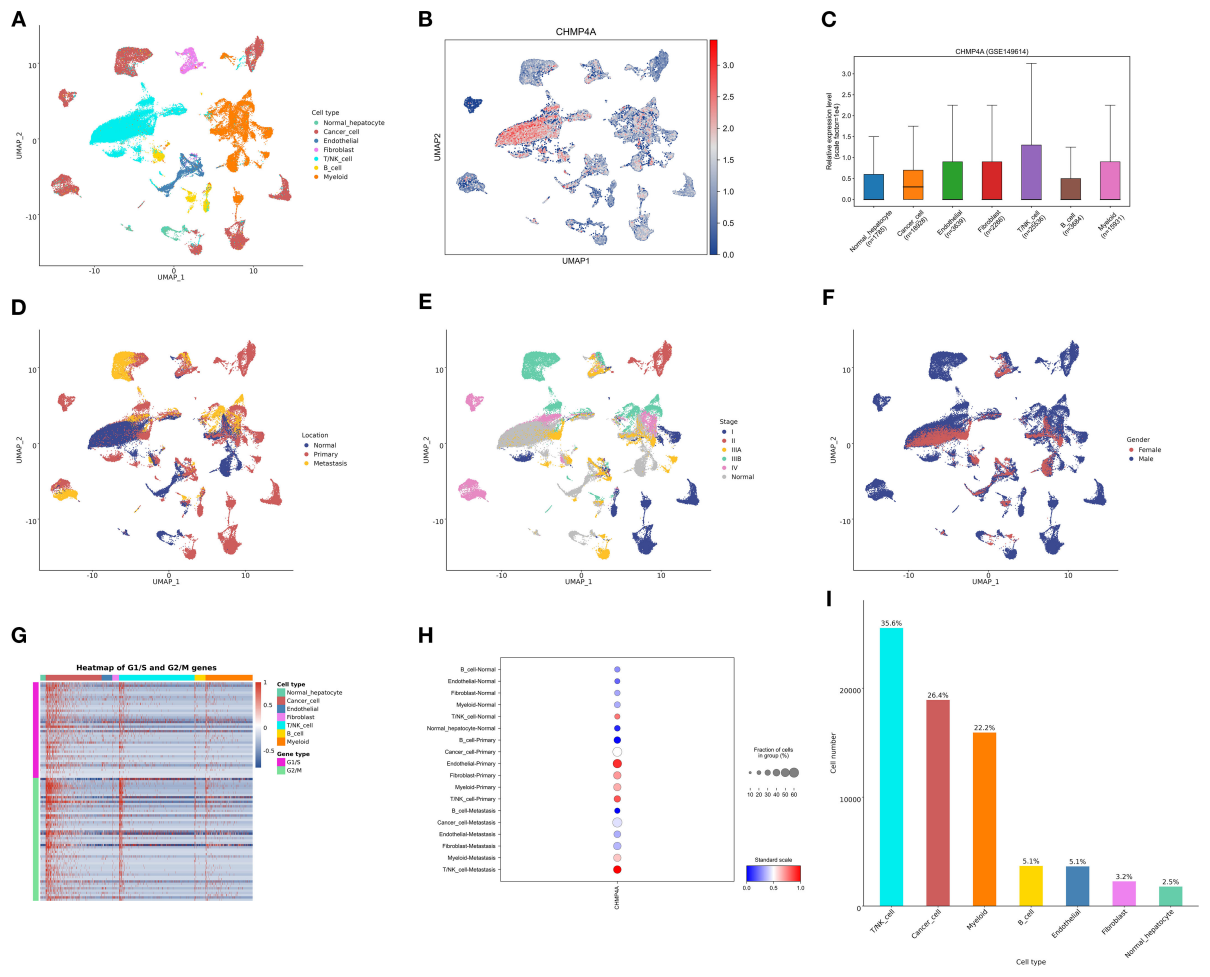
Single-cell analysis of CHMP4A in LIHC via scRNA-seq. **(A)** UMAP Visualization of Cell Type Distribution in LIHC. **(B)** UMAP Expression Profile of CHMP4A in LIHC. **(C)** Relative Expression Levels of CHMP4A Across Cell Types. **(D)** UMAP Visualization of Cell Distribution by Anatomical Location. **(E)** UMAP Visualization of Cell Distribution by Tumor Stage. **(F)** UMAP Visualization of Cell Distribution by Sex. **(G)** Heatmap of G1/S and G2/M Phase Transition Gene Expression Across Cell Types. **(H)** Expression Proportions of CHMP4A in Different Cell Types and Cancer Subtypes. **(I)** Statistics of Cell Number and Proportion for Each Cell Type. UMAP, Uniform Manifold Approximation and Projection; CHMP4A, Charged Multivesicular Body Protein 4A; CD4T_conv, Conventional CD4+ T cells; CD8T_typical, Typical CD8+ T cells; CD8T_exhausted, Exhausted CD8+ T cells; T_prolif, Proliferating T cells; Treg, Regulatory T cells; NK_cell, Natural Killer cell; B_cell, B lymphocyte; Mono/Macro, Monocyte/Macrophage; HCC, Hepatocellular Carcinoma; CC, Cholangiocarcinoma; G1/S, G1/S phase transition genes; G2/M, G2/M phase transition genes.

To examine the expression and cellular distribution of CHMP4A in LIHC at a single-cell resolution, we conducted an extensive single-cell RNA sequencing (scRNA-seq) analysis on LIHC tissues and adjacent normal liver tissues. Initially, UMAP dimensionality reduction was employed to visualize the cellular heterogeneity within the dataset, with cells color-coded according to their annotated cell types (**Figure 5A**). This analysis identified distinct clusters of major liver cell populations, including normal hepatocytes, cancer hepatocytes, endothelial cells, fibroblasts, B cells, T cells, and myeloid cells, thereby confirming the successful capture of diverse cellular components within the LIHC microenvironment. Subsequently, we assessed the expression pattern of CHMP4A across these cell clusters using a UMAP plot, where color intensity indicated normalized CHMP4A expression levels (**Figure 5B**). This visualization revealed heterogeneous CHMP4A expression, with notably high expression levels detected in specific subpopulations of certain immune cell clusters (T/NK cells) and cancer hepatocytes. To quantify this, we assessed the normalized expression levels of CHMP4A across various major cell types, confirming that T/NK cells and cancer cells exhibit the highest CHMP4A expression relative to other cell types (**Figure 5C**). Subsequently, we investigated the potential correlation between CHMP4A expression and tumor location by categorizing cells based on their origin, namely normal liver parenchyma, primary tumor, or metastasis, using UMAP analysis (**Figure 5D**). This investigation revealed that cells with high CHMP4A expression were predominantly localized within primary tumor and metastatic regions, with minimal expression observed in normal liver tissue. Additionally, we stratified the data by tumor stage (I, II, IIIA, IIIB, IV), which demonstrated a progressive increase in CHMP4A expression corresponding with advancing tumor stages, particularly notable in stages III and IV (**Figure 5E**). Moreover, gender-based stratification revealed no statistically significant differences in CHMP4A expression patterns between male and female patients, indicating that the upregulation of CHMP4A is not gender-specific (**Figure 5F**). To further investigate the biological context of CHMP4A upregulation, we constructed a heatmap illustrating the expression of genes associated with the G1/S and G2/M cell cycle transitions, alongside CHMP4A expression, across various cell types (**Figure 5G**). This heatmap demonstrated a strong positive correlation between CHMP4A expression and the expression of key cell cycle regulators, particularly in NK/T cells and cancer hepatocytes, suggesting that CHMP4A may play a role in promoting cell cycle progression in LIHC. Analysis of CHMP4A expression across different cell types and stages confirmed elevated expression levels in cancer cells and NK/T cells at all stages, with a marked increase observed in primary and metastatic stages (**Figure 5H**). Lastly, analysis of cell-type distribution within the dataset revealed that NK/T cells, cancer cells, and myeloid cells accounted for the largest proportions, a pattern consistent with the high CHMP4A expression observed in these cell types (**Figure 5I**). These findings collectively highlighted the pivotal role of CHMP4A within the cellular milieu of LIHC, especially in cancerous cells and NK/T cells, and its correlation with tumor progression and regulation of the cell cycle.

### Associations of CHMP4A expression levels with immune cell infiltration in LIHC

**Figure 6:**
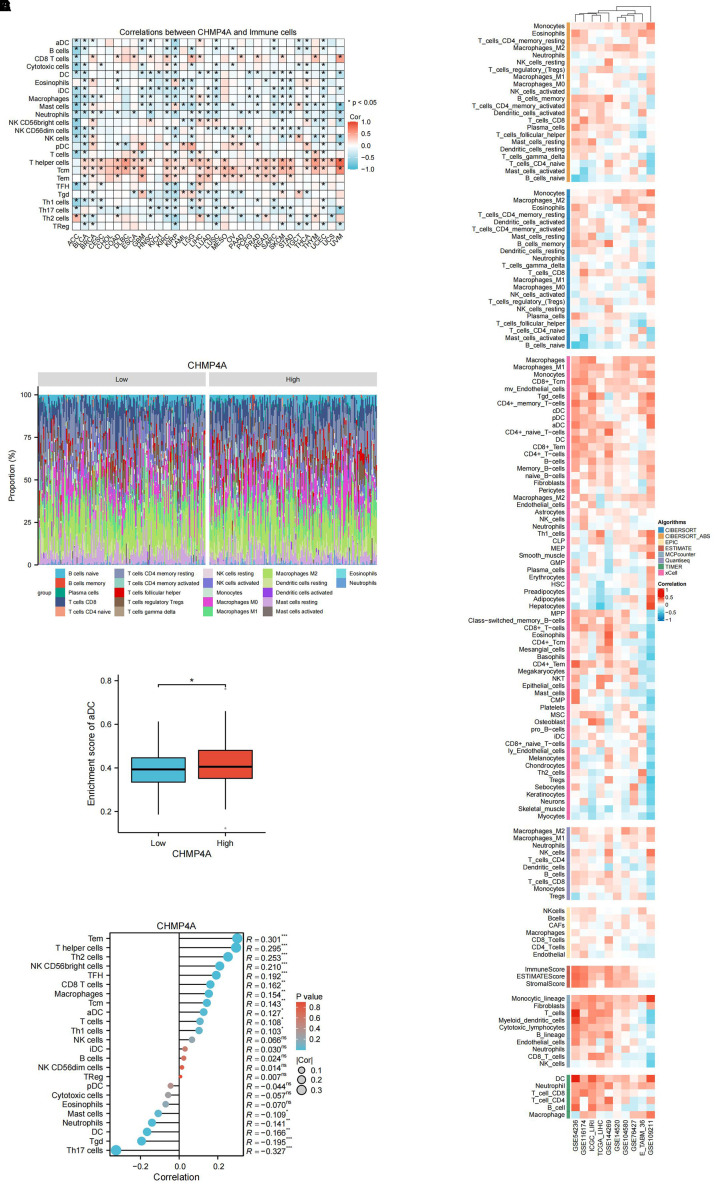
Integrative analysis of CHMP4A expression correlation with tumor microenvironment immune cells in LIHC. **(A)** Correlation of CHMP4A Expression with Immune Cells Across 33 Cancer Types. **(B)** Comparison of Immune Cell Proportions Stratified by CHMP4A Expression Levels (Low vs. High) in TCGA-LIHC. **(C)** Enrichment Score of Activated Dendritic Cells in LIHC Patients Stratified by CHMP4A Expression (Low vs. High). **(D)** Correlation Between CHMP4A Expression and Immune Infiltration in LIHC via ssGSEA Algorithm. **(E)** Correlation Between CHMP4A Expression and Immune Infiltration in LIHC Across Multiple Immune Infiltration Tools and Genomic Datasets. *P<0.05. CIBERSORT, cell-type identification by estimating relative subsets of RNA Transcripts; Cor, Pearson correlation coefficient; ESTIMATE, estimation of stromal and immune cells in malignant tumor tissues using expression data; LIHC, Liver Hepatocellular Carcinoma; Pval, p-value; TCGA, The Cancer Genome Atlas; xCell, cell type enrichment analysis tool.

In LIHC, the expression patterns of CHMP4A are significantly correlated with clinical characteristics, while tumor-infiltrating lymphocytes act as independent predictors of key clinical parameters, such as tumor stage, grade, and lymph node status. The tumor microenvironment, which includes tumor cells, stromal cells, and immune infiltrating cells, is crucial in cancer progression. To further investigate this relationship, we conducted an analysis using data from TCGA and GEO databases to examine the association between CHMP4A expression levels and immune cell infiltration in LIHC. A pan-cancer correlation analysis revealed that CHMP4A expression demonstrates cancer-type-specific associations with immune cell abundance (**Figure 6A**). Notably, although CHMP4A generally exhibited negative correlations with immune cell abundance profiles across 33 cancer types, it showed significant positive associations with T helper cells, central memory T cells (Tcm), effector memory T cells (Tem), and CD8 T cells. This trend was equally pronounced in LIHC. To further elucidate the differences in the immune landscape between LIHC patients exhibiting high versus low CHMP4A expression, we stratified the patients into “CHMP4A High” and “CHMP4A Low” cohorts based on the median expression levels of CHMP4A. We subsequently compared the relative proportions of various immune cell types (**Figure 6B**). The results indicated a significantly higher infiltration of T helper cells, Tem cells, and NK CD56bright cells in the CHMP4A High cohort. Conversely, regulatory T cells (Tregs), Th17 cells, and neutrophils were more prevalent in the CHMP4A Low cohort. Furthermore, the enrichment score for activated dendritic cells (aDCs) was elevated in the CHMP4A High cohort compared to the Low cohort (**Figure 6C**), suggesting a potential role for CHMP4A in facilitating dendritic cell activation. Furthermore, we utilized the single-sample Gene Set Enrichment Analysis (ssGSEA) algorithm to conduct a comprehensive analysis of immune cell infiltration patterns, uncovering significant associations between CHMP4A expression and 24 immune cell subtypes. Our findings indicated that CHMP4A exhibited moderate positive correlations with several key immune cells, including effector memory T cells (Tem, R=0.301, *P <*0.001), T helper cells (R=0.295, *P <*0.001), Th2 cells (R=0.253, *P*<0.001), and NK CD56bright cells (R=0.210, *P*<0.001), suggesting their potential co-enrichment within the tumor microenvironment. Additionally, weaker yet statistically significant positive correlations were observed with follicular helper T cells (TFH, R=0.192, *P*<0.001), CD8+ T cells (R=0.162, *P*<0.01), macrophages (R=0.154, *P*<0.01), central memory T cells (Tcm, R=0.154, *P*<0.01), and aDCs (R=0.127, *P*<0.05). Conversely, we identified moderate negative correlations with Th17 cells (R = -0.327, *P*<0.001), gamma delta T cells (Tgd, R = -0.195, *P <*0.001), dendritic cells (DC, R = -0.166, *P*<0.01), and neutrophils (R = -0.141, *P*<0.01), indicating potential patterns of mutual exclusion (**Figure 6D**). To substantiate these findings, we employed a suite of immune infiltration analysis tools, including EPIC, ESTIMATE, TIMER, MCP-Counter, QuanTIseq, and XCELL, across nine independent genomic datasets (GSE54236, GSE116174, ICGC-LIRI, GSE144269, GSE14520, GSE104580, GSE76427, E_TABM_36, and GSE109211). This comprehensive methodological approach consistently characterized the tumor immune microenvironment across various computational platforms, thereby robustly affirming the reliability of our observations (**Figure 6E**).

### Analysis of immune regulatory genes, TMB, MSI, and immune checkpoints related to CHMP4A in LIHC

**Figure 7:**
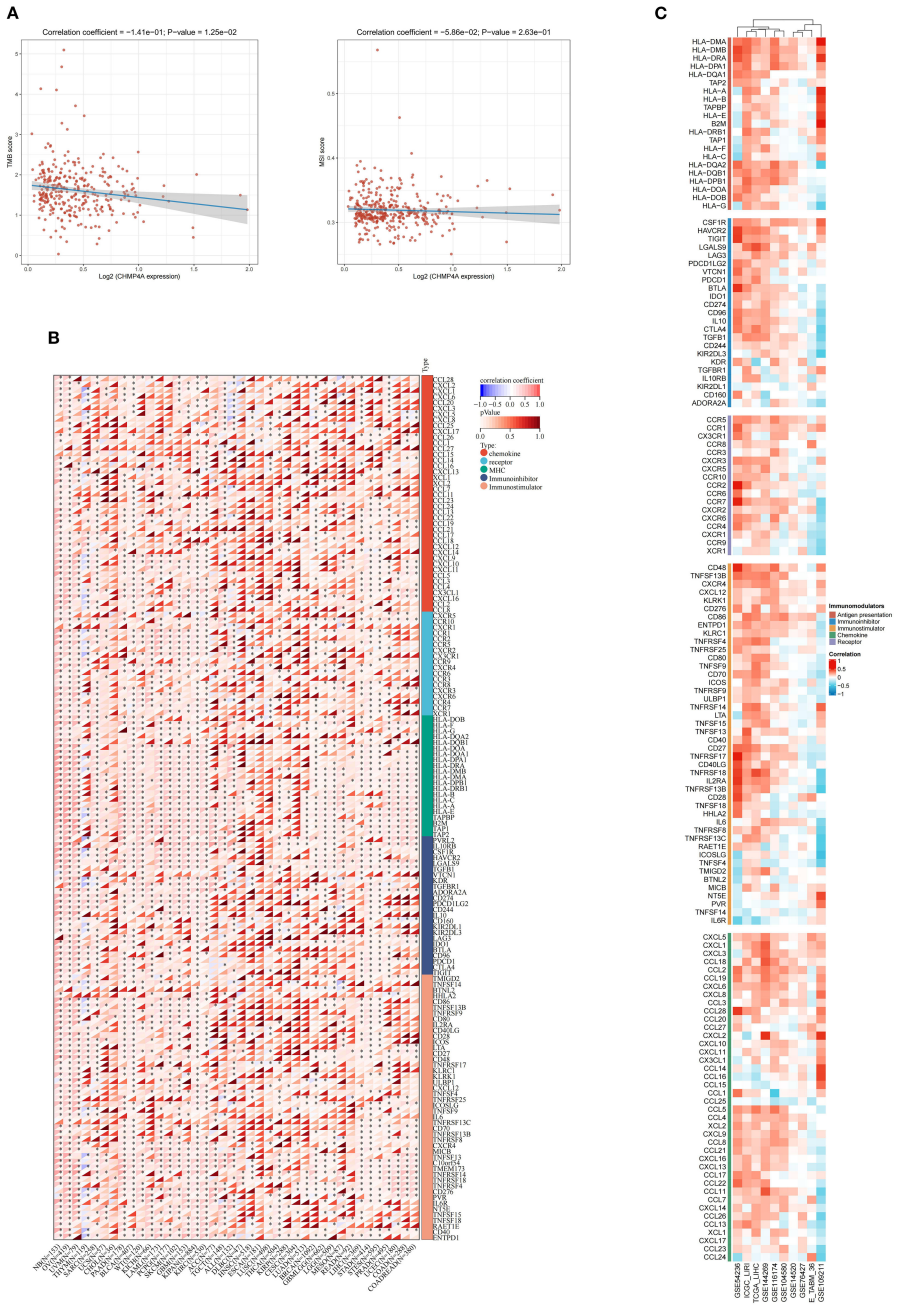
Integrated analysis of associations between CHMP4A expression and immune-related genes. **(A)** Associations of CHMP4A Expression Levels with TMB and MSI in LIHC. **(B)** Pan-Cancer Associations Between CHMP4A Expression and Immune-Related Genes. **(C)** Associations Between CHMP4A Expression and Immune-Related Genes in LIHC Across Multiple Immune Infiltration Tools and Genomic Datasets. *P<0.05, **P<0.01, ***P<0.001. LIHC, Liver Hepatocellular Carcinoma; Pearson, Pearson correlation coefficient; Cor, Correlation coefficient.

Evidence indicates that TMB and MSI-H serve as pertinent biomarkers for predicting tumor response to immune checkpoint inhibitors (ICI). However, TMB exhibits considerable variability among MSI-H tumors. In our study, we observed a significant inverse correlation between elevated CHMP4A expression and TMB (R = -0.141, *P*=0.0125), while no significant association was found with MSI (R = -0.0586, *P*=0.263) (**Figure 7A**). The efficacy of immune checkpoint blockade (ICB) therapy is contingent upon a multitude of factors, including immune cell infiltration, the presence of immune checkpoints, and the expression of immune regulatory genes. To elucidate these complex interactions, we conducted an analysis of CHMP4A mRNA expression levels in conjunction with various immune-related genes-encompassing chemokines, chemokine receptors, major histocompatibility complex (MHC) molecules, immunoinhibitors, and immunostimulators—across 32 distinct cancer types utilizing data from TCGA (**Figure 7B**). This comprehensive methodology provides insights into the interactions between CHMP4A and critical immune components within diverse tumor microenvironments. CHMP4A exhibited extensive positive associations with immune-related genes across various malignancies, with distinct cancer-specific patterns emerging, except in thymoma (THYM) and acute lymphoblastic leukemia (ALL), where inverse relationships were observed. In LIHC, CHMP4A expression demonstrated almost universal positive correlations with immune-related markers, particularly immune checkpoint molecules such as TIM3 (HAVCR2), LGALS9, CSF1R, TGFB1, and VTCN1 (**Figure 7B**). To substantiate these findings, we performed comprehensive correlation analyses between CHMP4A expression and 137 immune modulators, categorized into five functional groups: antigen presentation molecules, chemokines, inhibitory immune checkpoints, stimulatory immune checkpoints, and immune receptors. These analyses utilized nine independent datasets, including GSE54236, ICGC_LIRI, etc (**Figure 7C**). The results consistently indicated positive associations with immunosuppressive checkpoints, with TIM3 exhibiting the strongest correlation, followed by TIGIT and BTLA. Notably, LGALS9, functioning as a ligand for TIM3, exhibited a robust positive correlation with CHMP4A, which may suggest a role in mechanisms of T-cell dysfunction. The consistent positive associations with immune-related factors across various datasets reinforced the reliability of these findings, indicating that CHMP4A plays a pivotal role in influencing the immune landscape within the LIHC tumor microenvironment.

### Response to immunotherapy and drug sensitivity

**Figure 8:**
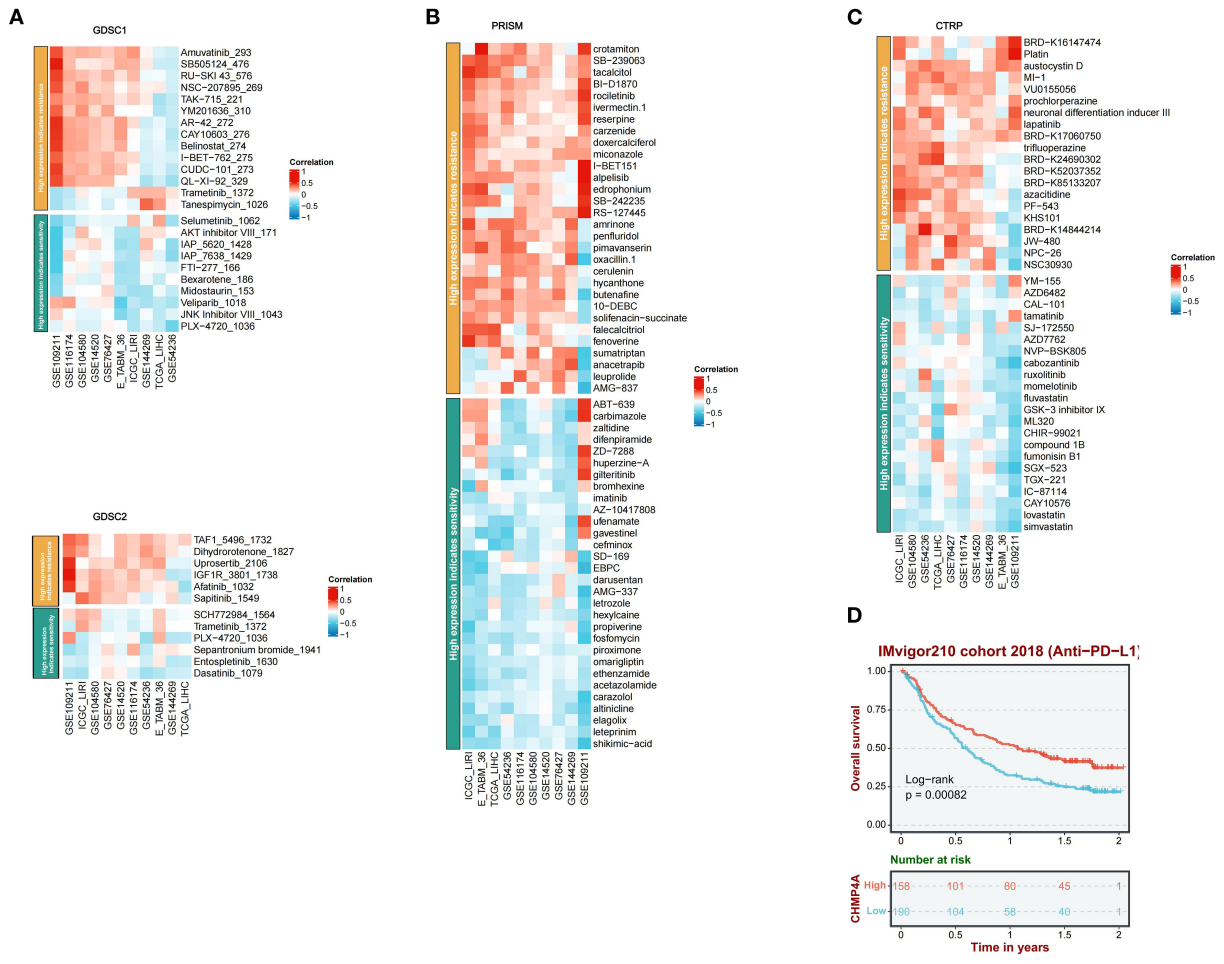
A comprehensive analysis of the correlation between CHMP4A expression and drug response across multiple databases, along with its association with survival outcomes. **(A)** Correlation between CHMP4A expression and drug resistance/sensitivity in the GDSC1 and GDSC2 datasets. **(B)** Correlation between CHMP4A expression and drug resistance/sensitivity in the PRISM dataset. **(C)** Correlation between CHMP4A expression and drug resistance/sensitivity in the CTRP database. **(D)** Overall survival analysis of the IMvigor210 cohort 2018 (Anti-PD-L1). CTRIP, Cancer Therapeutics Response Portal; PRISM, Preclinical Repurposing of Medicines; GDSC1/GDSC2, Genomics of Drug Sensitivity in Cancer 1/2; Anti-PD-L1, Anti-Programmed Death-Ligand 1; Log-rank, Log-rank test; Number at risk, Number of patients at risk at each time point.

A comprehensive pharmacogenomic analysis conducted across nine LIHC datasets (GSE54236, GSE116174, ICGC-LIRI, GSE144269, GSE14520, GSE104580, GSE76427, E-TABM-36, GSE109211) and four drug screening platforms (CTRP, PRISM, GDSC1, GDSC2) had elucidated that increased expression of CHMP4A was associated with dual therapeutic outcomes, specifically immunotherapy resistance and context-dependent sensitivity to targeted agents (**Figures 8A–C**). Elevated CHMP4A expression demonstrated a strong correlation with resistance to a wide range of therapeutic classes, including kinase inhibitors (Amuvatinib_293, a c-Kit/PDGFR inhibitor; SB505124_476, a TGF-βR kinase inhibitor; RU-SKI 43_576, an mTOR inhibitor; BI-D1870, an RSK kinase inhibitor; rociletinib, an EGFR kinase inhibitor; PLX-4720_1036, a BRAF inhibitor; Entospletinib_1630, a SYK kinase inhibitor; Dasatinib_1079, an SRC/ABL kinase inhibitor), metabolic modulators (Dihydrorotenone_1827, a mitochondrial complex I inhibitor; crotamiton, an ammonia scavenger; tacalcitol, a vitamin D analog), and cell cycle regulators (TAF1_5496_1732, a CDK inhibitor; SB-239063, a p38 MAPK inhibitor; Platin, a cisplatin analog; austocystin D, a cytotoxic marine compound; MI-1, an HDAC inhibitor). In contrast, tumors with high CHMP4A expression exhibited increased sensitivity to cholesterol-lowering agents (lovastatin, simvastatin, HMG-CoA reductase inhibitors), kinase pathway inhibitors (JNK Inhibitor VIII_1043, a JNK kinase inhibitor), neuroimmunomodulators (altinicline, an α4β2 nicotinic receptor agonist), steroidal antagonists (elagolix, a GnRH antagonist, leteprinim, a progesterone antagonist), tyrosine kinase activators (CAY10576, an EPHB2 agonist), and antimicrobial precursors (shikimic acid, a shikimate pathway modulator). Survival analysis within the IMvigor210 anti-PD-L1 immunotherapy cohort further revealed that patients receiving anti-PD-L1 immunotherapy with high CHMP4A expression demonstrated significantly improved overall survival compared to those with low expression levels, thereby validating CHMP4A as a potential predictive biomarker for resistance to immune checkpoint inhibitors (**Figure 8D**). Collectively, these findings established CHMP4A as a dual-functional biomarker that not only predicted resistance to immunotherapy but also influenced differential drug sensitivity. This suggested actionable strategies for managing CHMP4A-high LIHC, emphasizing the use of statins, JNK inhibitors, and EPHB2 agonists, while avoiding resistance-associated kinase and cell cycle-targeting agents.

### Downregulation of CHMP4A attenuated proliferation, migration, and invasion in LIHC cells

**Figure 9:**
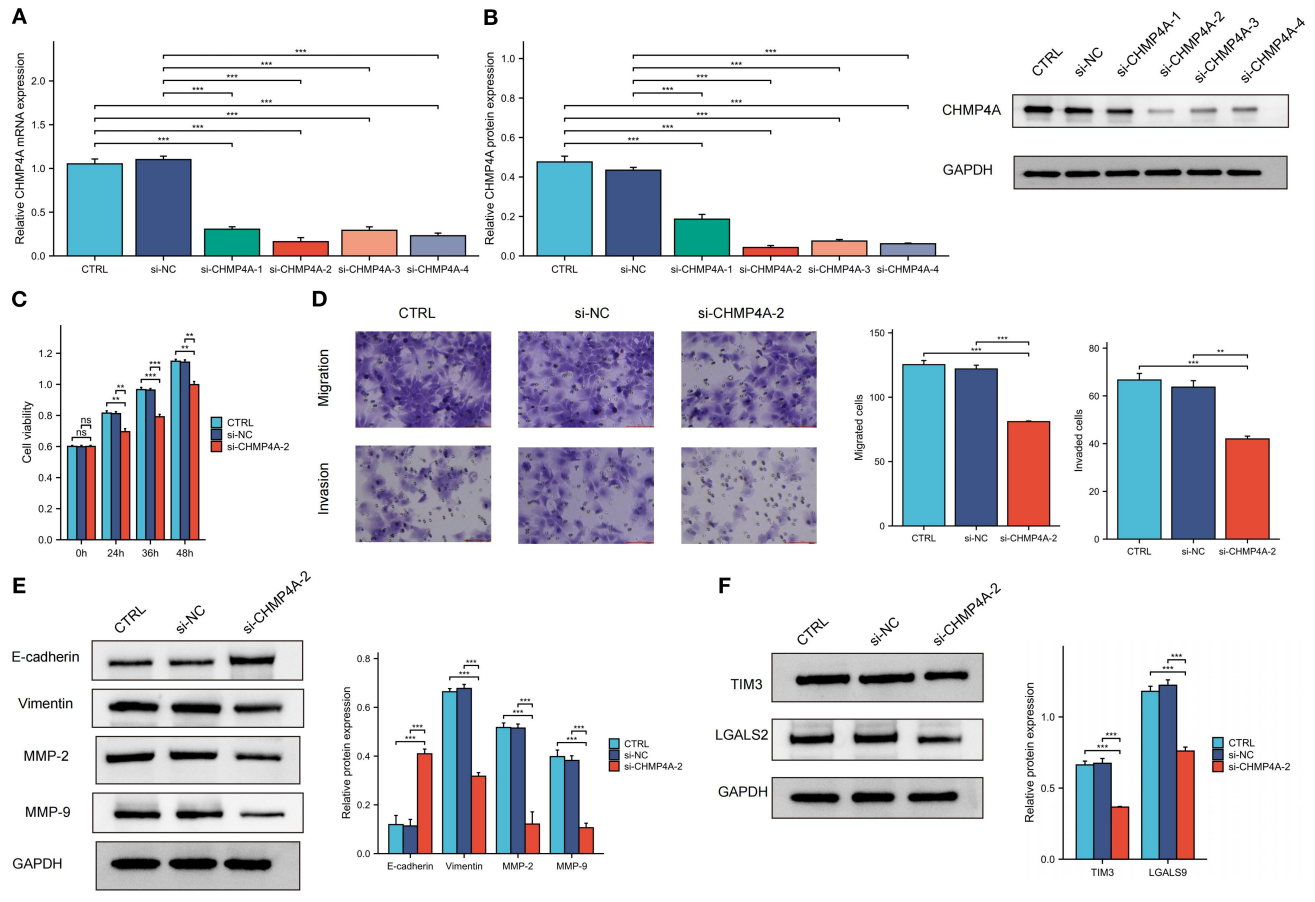
The knockdown of CHMP4A inhibited the proliferation, migration, and invasion of LIHC cells, as well as the TIM-3/Galectin-9 signaling pathway. **(A, B)** RT-qPCR and Western blot validation of CHMP4A silencing efficiency using siRNAs (si-CHMP4A-1 to -4) with GAPDH as a loading control. **(C)** CCK-8 cell viability assay showing reduced proliferation of HEP 3B2.1–7 cells after CHMP4A knockdown (si-CHMP4A-2). **(D)** Transwell assay revealing a reduction in the migratory and invasive capabilities of HEP 3B2.1–7 cells following the knockdown of CHMP4A. **(E)** Immunoblot analysis of EMT markers and TIM-3 axis components showing upregulation of E-cadherin and downregulation of Vimentin, MMP-2, and MMP-9 in si-CHMP4A-treated cells. **(F)** Immunoblot analysis of TIM-3 axis components showing downregulation of HAVCR2 (TIM-3) and LGALS9 in si-CHMP4A-treated cells. **P<0.01, ***P<0.001. CTRL, control untreated; si-NC, negative control siRNA; si-CHMP4A, CHMP4A-targeting siRNA; E-cadherin, epithelial cadherin; MMP-2/9, matrix metalloproteinase-2/9; HAVCR2, hepatitis A virus cellular receptor 2 (TIM-3); LGALS9, lectin galactoside-binding soluble 9 (Galectin-9).

To investigate CHMP4A’s biological role in hepatocellular carcinoma progression, we performed multiple *in vitro* experiments using CHMP4A-silenced HEP 3B2.1–7 cells. We introduced four distinct siRNA sequences targeting CHMP4A into these cells, confirming the effective suppression of CHMP4A expression via western blotting and quantitative PCR, which revealed significant differences compared to control groups and cells treated with si-NC (**Figures 9A, B**). After thorough evaluation, the most effective siRNA (si-CHMP4A-2) was selected for further functional analysis. Assessment of cell proliferation using the CCK-8 assay indicated that CHMP4A deficiency markedly reduced the proliferative capacity of HEP 3B2.1–7 cells (**Figure 9C**). Furthermore, transwell assays demonstrated that CHMP4A silencing significantly diminished the migratory and invasive capabilities of these cells (**Figure 9D**). In light of the well-established role of epithelial-mesenchymal transition (EMT) in promoting cancer cell dissemination, we investigated the principal molecular markers associated with this process. EMT is typified by a decrease in the expression of epithelial adhesion molecules, such as E-cadherin, alongside an increase in mesenchymal proteins, including vimentin and matrix metalloproteinases (MMP-2 and MMP-9), which collectively enhance cellular motility and invasion (39–41). Protein expression profiling revealed that the knockdown of CHMP4A resulted in a significant increase in E-cadherin levels, while concurrently reducing the expression of vimentin, MMP-2, and MMP-9 (**Figure 9E**). These findings provided compelling experimental evidence for the role of CHMP4A in advancing hepatocellular carcinoma progression by modulating EMT-associated molecular pathways that regulate tumor cell proliferation and metastasis.

### CHMP4A silencing reduced TIM3 and LGALS9 expression in LIHC cells

In the aforementioned text, our study conducted a systematic examination of the relationship between CHMP4A expression patterns and the characteristics of immune cell infiltration within tumor microenvironments. The analysis identified a significant positive correlation between CHMP4A levels and several key immunosuppressive checkpoint molecules. Notably, a strong positive correlation in expression was observed between CHMP4A and both TIM3 (HAVCR2) and its cognate ligand galectin-9 (LGALS9). To mechanistically validate these bioinformatic findings, we conducted targeted CHMP4A knockdown experiments in the HEP 3B2.1–7 LIHC cell line. The experimental findings revealed that the silencing of CHMP4A led to a marked decrease in the expression levels of TIM3 and LGALS9 in LIHC cells, suggesting a potential regulatory role in TIM3/LGALS9 expression. However, the downstream immune effects of this regulation have yet to be fully elucidated (**Figure 9F**).

## Discussion

In LIHC pathophysiology, various transporters are crucial to its development. The ESCRT-III family, including CHMPs, is vital for processes like membrane remodeling and protein degradation, maintaining cellular balance (10, 12). In LIHC, ESCRT-III is linked to tumor growth and spread. Studies show CHMPs are highly expressed in liver tumors, associated with poor outcomes and drug resistance, highlighting their potential as therapeutic targets (11). Emerging evidence underscores CHMP4A’s role in LIHC pathogenesis, making it a promising target for cancer research. In the liver tumor microenvironment, immune regulation is key to disease progression and treatment outcomes. CHMP4A, known for its role in endocytic sorting and vesicular transport, has shown oncogenic relevance, particularly in breast cancer models where it aids CD8+ T-cell recruitment and inhibits tumor growth via LSD1-mediated interferon-β pathways (15, 42). These findings suggest that CHMP4A’s ability to modulate the immune system is particularly relevant in LIHC, where tumor immune evasion often hinders treatment. CHMP4A’s role in promoting cytotoxic T lymphocyte infiltration holds promise for developing LIHC immunotherapies (15). Genomic analyses show that CHMP4A-related pyroptotic pathway polymorphisms can predict clinical outcomes in various cancers, including some head and neck squamous cell carcinomas (16). This highlights CHMP4A’s importance in immune response modulation and its potential as a prognostic marker.

This research showed that CHMP4A mRNA was significantly higher in various cancer tissues, particularly in tumor tissues of TCGA-LIHC samples compared to normal tissues. The pan-cancer analysis enhanced the understanding of CHMP4A by contextualizing its differential expression and prognostic significance across 33 cancer types from TCGA, and it highlighted the gene’s notable oncogenic role in LIHC compared to other cancer types. Higher tumor grades (G3/G4) had more CHMP4A expression than lower grades (G1/G2), while larger tumors showed less. Viral hepatitis was linked to reduced CHMP4A levels, and Sorafenib non-responders had higher expression. The HPA database and immunohistochemistry confirmed increased CHMP4A in LIHC tissues. High CHMP4A expression in LIHC correlated with poorer survival, as shown by ICGC and GEO datasets. Multivariate analysis identified CHMP4A overexpression and advanced T-stage as independent predictors of poor survival, highlighting its prognostic significance. This finding delineated the multifaceted role of CHMP4A in LIHC, consistent with existing studies that highlighted its oncogenic function across various cancers. Based on this, further investigation into its pro-oncogenic mechanisms in LIHC progression was essential. A promising direction could have involved exploring CHMP4A’s interactions with other tumor microenvironment components; specifically, it might have interacted with signaling pathways that governed tumor cell proliferation, invasion, and metastasis.

GSEA revealed that high CHMP4A expression in LIHC enhances immune-related processes, while KEGG and Hallmark analyses linked it to key cancer pathways such as actin cytoskeleton remodeling and phagocytosis. Protein interaction networks identified CHMP4A as a hub in vesicular trafficking and membrane dynamics via interactions with ESCRT-III and CHMP family proteins, implying it contributes to LIHC by disrupting intracellular transport and immune signaling. Notably, consistent with its association with oncogenic processes from enrichment analyses, CHMP4A was likely associated with actin cytoskeleton dynamics—critical for cell motility, invasion, and cancer metastasis. Our DNA methylation analysis indicated hypomethylation of the CHMP4A promoter in LIHC tissues compared to normal liver, which may enhance CHMP4A transcription and lead to increased mRNA and protein expression. Bioinformatics approaches further suggested several transcription factors that may regulate CHMP4A, and experimental validation of these candidates is underway. Single-cell RNA sequencing revealed heterogeneous CHMP4A expression, with particularly high levels in T cells, NK cells, and malignant hepatocytes. CHMP4A was upregulated in both primary and metastatic tumors, and its expression correlated positively with advancing tumor stage. Moreover, CHMP4A expression was positively correlated with key cell cycle regulators in NK/T cells and cancer cells, suggesting a potential role in promoting cell cycle progression in LIHC. Given its elevated expression in immune subsets, we propose that CHMP4A may contribute to immune activation—possibly by regulating cytokine production or enhancing cytotoxic function in T and NK cells within the tumor microenvironment.

The tumor immune microenvironment critically shapes cancer progression and metastasis. Characterizing tumor-infiltrating immune cells could therefore advance therapeutic strategies and enhance response to immune checkpoint inhibitors. Due to the typically poor immune response and limited efficacy of immunotherapy in LIHC, we systematically evaluated CHMP4A expression in relation to immune infiltration patterns both in pan-cancer analyses and specifically in LIHC. Our pan-cancer analysis revealed context-specific roles of CHMP4A, with particularly strong associations between high CHMP4A expression, poor prognosis, and immune infiltration in LIHC. Interestingly, while CHMP4A expression generally correlated with immunosuppressive features across cancers, in LIHC it showed positive associations with certain anti-tumor lymphocytes—including T helper cells, Tem and NK CD56bright cells. These patterns, consistent across nine independent cohorts, indicated that CHMP4A might have helped establish an immune-permissive microenvironment by promoting dendritic cell activation and cytotoxic cell recruitment. Pan-cancer analysis further indicated strong positive correlations between CHMP4A and immune-related genes, particularly immunosuppressive checkpoints such as TIM3 (HAVCR2), LGALS9, TIGIT, and BTLA. These results suggested that CHMP4A may promote an immunosuppressive environment in LIHC, potentially via the TIM3/LGALS9 axis and T-cell exhaustion mechanisms. The TIM3/LGALS9 signaling axis is known for its role in immune evasion by cancer cells, which use it to suppress anti-tumor immune responses, worsening patient outcomes (43, 44). To investigate the link between CHMP4A and the TIM3/LGALS9 pathway, we knocked down CHMP4A in HEP 3B2.1–7 liver cancer cells. Silencing CHMP4A reduced the expression of TIM3 and LGALS9, suggesting a regulatory role for CHMP4A in this immune checkpoint axis. We hypothesized that CHMP4A might influence TIM3/LGALS9 through endosomal regulation, transcriptional crosstalk, or TIM3-related T cell exhaustion. However, the underlying mechanisms, particularly regarding how CHMP4A might affect protein stability, remained elusive. Further studies using co-culture systems or xenograft models were needed to clarify how CHMP4A affects immune-related pathways. Integrating our immunotherapy and drug sensitivity data, CHMP4A was established as a bifunctional biomarker that may predict immunotherapy resistance and could modulate therapeutic response, thereby offering potential strategies for personalized LIHC management.

The correlation between aberrant CHMP4A expression and poor prognosis in LIHC patients prompted further investigation into its biological functions. To systematically assess its role in LIHC progression, we performed *in vitro* experiments using CHMP4A-knockdown models in HEP 3B2.1–7 cells. Depletion of CHMP4A significantly suppressed cell proliferation in CCK-8 assays and reduced metastatic ability in Transwell migration and invasion assays. Given the importance of EMT in metastasis, we examined its markers and found that CHMP4A knockdown increased E-cadherin expression and decreased vimentin, MMP-2, and MMP-9 levels. These results suggested that CHMP4A promoted LIHC development by enhancing proliferation and EMT-mediated metastasis, although the precise mechanisms remained unclear.

While our study integrated bioinformatics, *in vitro*, and tissue-based approaches, the molecular mechanisms involved require further elucidation. Subsequent experiments will employ multiple cell lines and animal models for *in vitro* and *in vivo* validation, alongside more comprehensive mechanistic investigations.

In conclusion, our extensive investigation clarified the complex role of CHMP4A in the pathogenesis of LIHC and its relationship with the tumor immune microenvironment. We demonstrated that increased expression of CHMP4A not only accelerated tumor progression but might also have been correlated with changes in immune cell infiltration patterns, indicating a potential association between CHMP4A and the remodeling of immune contexture. Knockdown of CHMP4A inhibited LIHC cell proliferation and migration, increased E-cadherin expression, and decreased levels of vimentin, MMP-2, MMP-9, and the immune checkpoint factors TIM3/LGALS9. These findings underscored the dual potential of CHMP4A as both a predictive biomarker and a promising therapeutic target in LIHC management. Clinically, assessment of CHMP4A could enable personalized treatment strategies and improve patient outcomes.

## Data Availability

The original contributions presented in the study are included in the article/**Supplementary Material**. Further inquiries can be directed to the corresponding authors.

## Supplementary material

Supplementary Figure 1
**(A)** Chromosomal localization of CHMP4A in the human genome. **(B)** Genomic architecture of CHMP4A and its flanking regions.

Supplementary Figure 2
**(A)** The GSEA-GO enrichment profile of CHMP4A in the context of immune regulation, as indicated by the enrichment score. **(B)** The GSEA-KEGG enrichment profile of CHMP4A in the context of immune regulation, as indicated by the enrichment score; **(C)** Hallmark gene set enrichment of CHMP4A in LIHC. * *P*<0.05, ** *P*<0.01, *** *P*<0.001, **** *P*<0.0001. LIHC, Liver Hepatocellular Carcinoma; CHMP4A, Charged Multivesicular Body Protein 4A; ES, Enrichment Score; GSEA, Gene Set Enrichment Analysis; GO, Gene Ontology; KEGG, Kyoto Encyclopedia of Genes and Genomes; CHMP4A, Charged Multivesicular Body Protein 4A; TCGA, The Cancer Genome Atlas.

## Glossary

CHMP4A: Charged Multivesicular Body Protein 4A
LIHC: liver hepatocellular carcinoma
AFP: Alpha-fetoprotein
TCGA: The Cancer Genome Atlas
GEO: Gene Expression Omnibus
ICGC: International Cancer Genome Consortium
IHC: immunohistochemistry
HPA: Human Protein Atlas
AUC: Area Under Curve
CI: Confidence Interval
ROC: Receiver Operating Characteristic
TPM: Transcripts Per Million
CC: Cholangiocarcinoma
CNV: Copy Number Variation
SNV: Single Nucleotide Variant
WES: Whole Exome Sequencing
CpG: Cytosine-phosphate-Guanine dinucleotide
HCC: hepatocellular carcinoma
TNM: Tumor-Node-Metastasis staging system
ES: Enrichment Score
GSEA: Gene Set Enrichment Analysis
CIBERSORT: cell-type identification by estimating relative subsets of RNA Transcripts
Cor: Pearson correlation coefficient
ESTIMATE: estimation of stromal and immune cells in malignant tumor tissues using expression data
xCell: cell type enrichment analysis tool
Pearson: Pearson correlation coefficient
Cor: Correlation coefficient
UMAP: Uniform Manifold Approximation and Projection
CD4T_conv: Conventional CD4+ T cells
CD8T_typical: Typical CD8+ T cells
CD8T_exhausted: Exhausted CD8+ T cells
T_prolif: Proliferating T cells
Treg: Regulatory T cells
NK_cell: Natural Killer cell
B_cell: B lymphocyte
Mono/Macro: Monocyte/Macrophage
HCC: Hepatocellular Carcinoma
CC: Cholangiocarcinoma
G1/S: G1/S phase transition genes
G2/M: G2/M phase transition genes
CTRP: Cancer Therapeutics Response Portal
PRISM: Preclinical Repurposing of Medicines
GDSC1/GDSC2: Genomics of Drug Sensitivity in Cancer 1/2
Anti-PD-1: Anti-Programmed Cell Death Protein 1
Anti-CTLA-4: Anti-Cytotoxic T-Lymphocyte-Associated Protein 4
Log-rank: Log-rank test
Number at risk: Number of patients at risk at each time point
CTRL: control untreated
si-NC: negative control siRNA
si-CHMP4A: CHMP4A-targeting siRNA
E-cadherin: epithelial cadherin
MMP-2/9: matrix metalloproteinase-2/9
HAVCR2: hepatitis A virus cellular receptor 2 (TIM-3)
LGALS9: lectin galactoside-binding soluble 9 (Galectin-9)
LUAD: Lung adenocarcinoma
LUSC: Lung squamous cell carcinoma
TMB: Tumor mutation burden;MSI, Microsatellite instability
GTEx: Genotype-Tissue Expression databases
OS: overall survival
PFS: progression-free survival
RFS: Relapse-Free Survival
ROC: receiver operating characteristic
AUCs: area under the curves
C-index: consistency index
KM plotter: Kaplan-Meier plotter
IOD: integrated optical density
FC: fold-change
HR: Hazard ratio
siRNA: small interfering RNA
OD: optical density
GBM: Glioblastoma
GBMLGG: Glioblastoma and Lower Grade Glioma
LGG: Lower Grade Glioma
BRCA: Breast Cancer
KIRP: Kidney Papillary Cell Carcinoma
STAD: Stomach Adenocarcinoma
HNSC: Head and Neck Squamous Cell Carcinoma
KIRC: Kidney Renal Clear Cell Carcinoma
LIHC: Liver Hepatocellular Carcinoma
PAAD: Pancreatic Adenocarcinoma
TILs: tumor-infiltrating lymphocytes
TME: tumor microenvironment
ICI: Immune checkpoint inhibitors
