# Supplementary figures and images for: CHMP4A in hepatocellular carcinoma: exploring its role in tumor progression, immune modulation, and potential link to TIM3 checkpoint
Source: Front Immunol. 2025 Oct 2;16:1682724. doi: 10.3389/fimmu.2025.1682724 (PMC12528155; doi:10.3389/fimmu.2025.1682724)

A

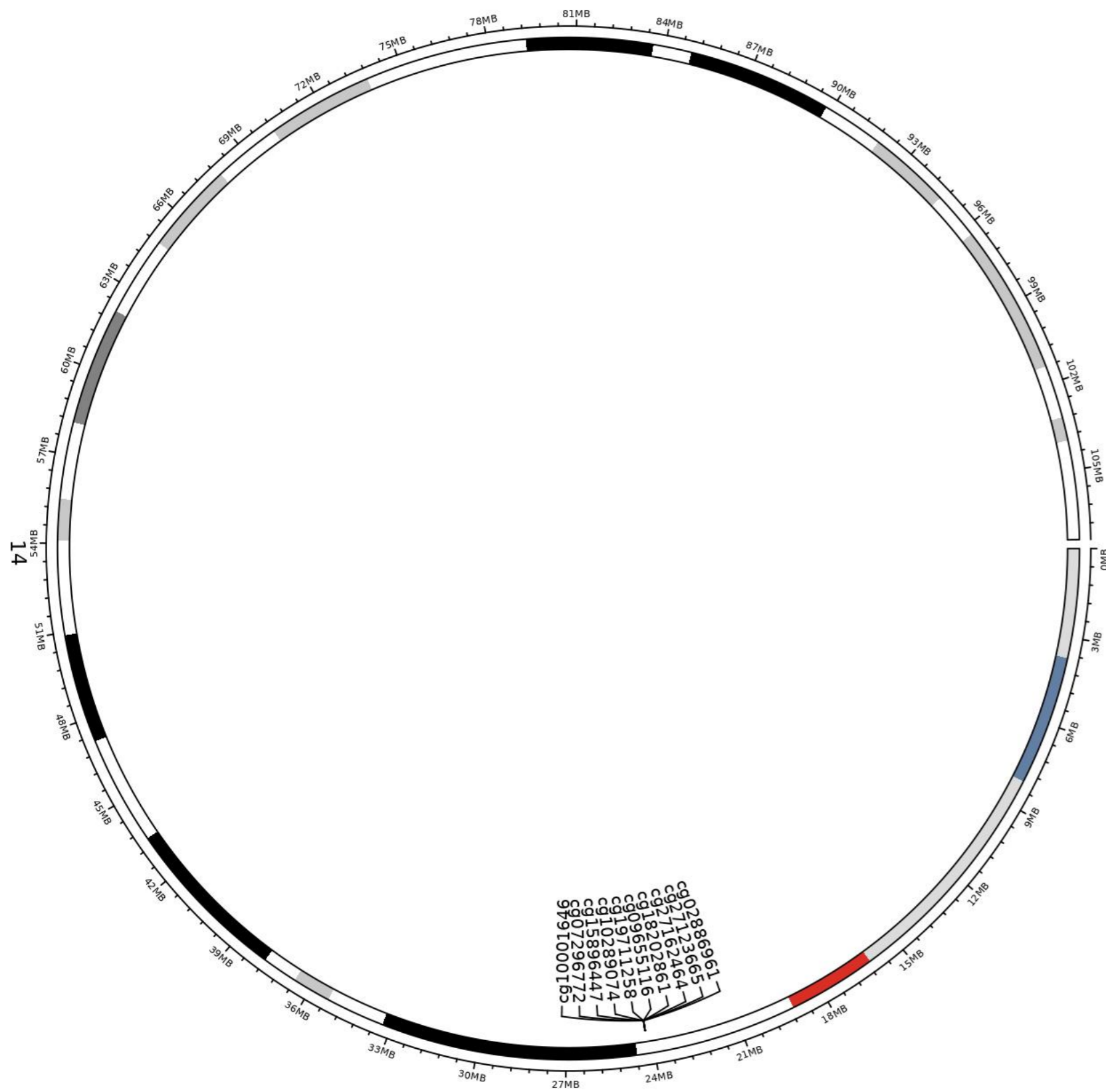

B

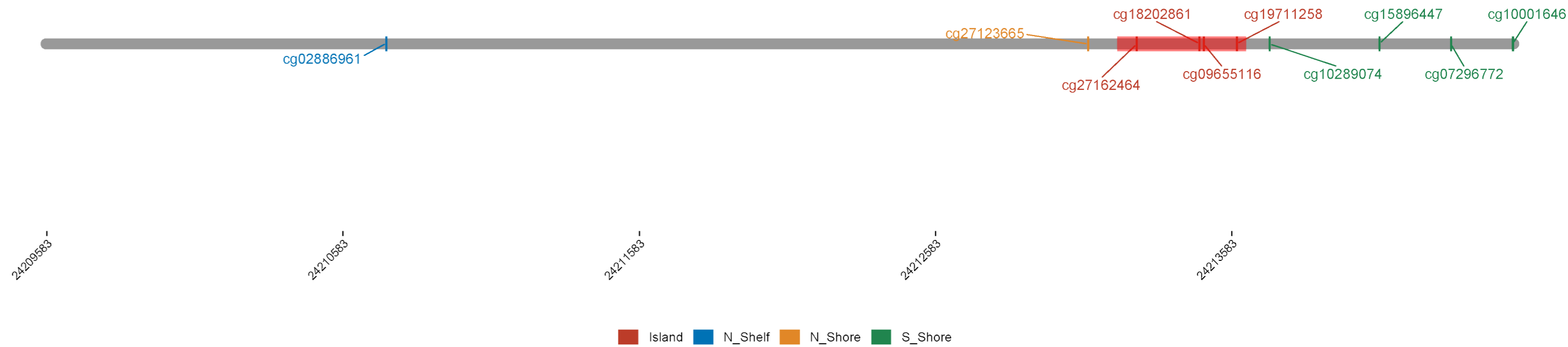

Supplement: Supplementary Figure 1 — (A) Chromosomal localization of CHMP4A in the human genome. (B) Genomic architecture of CHMP4A and its flanking regions. [file Image1.pdf]

A

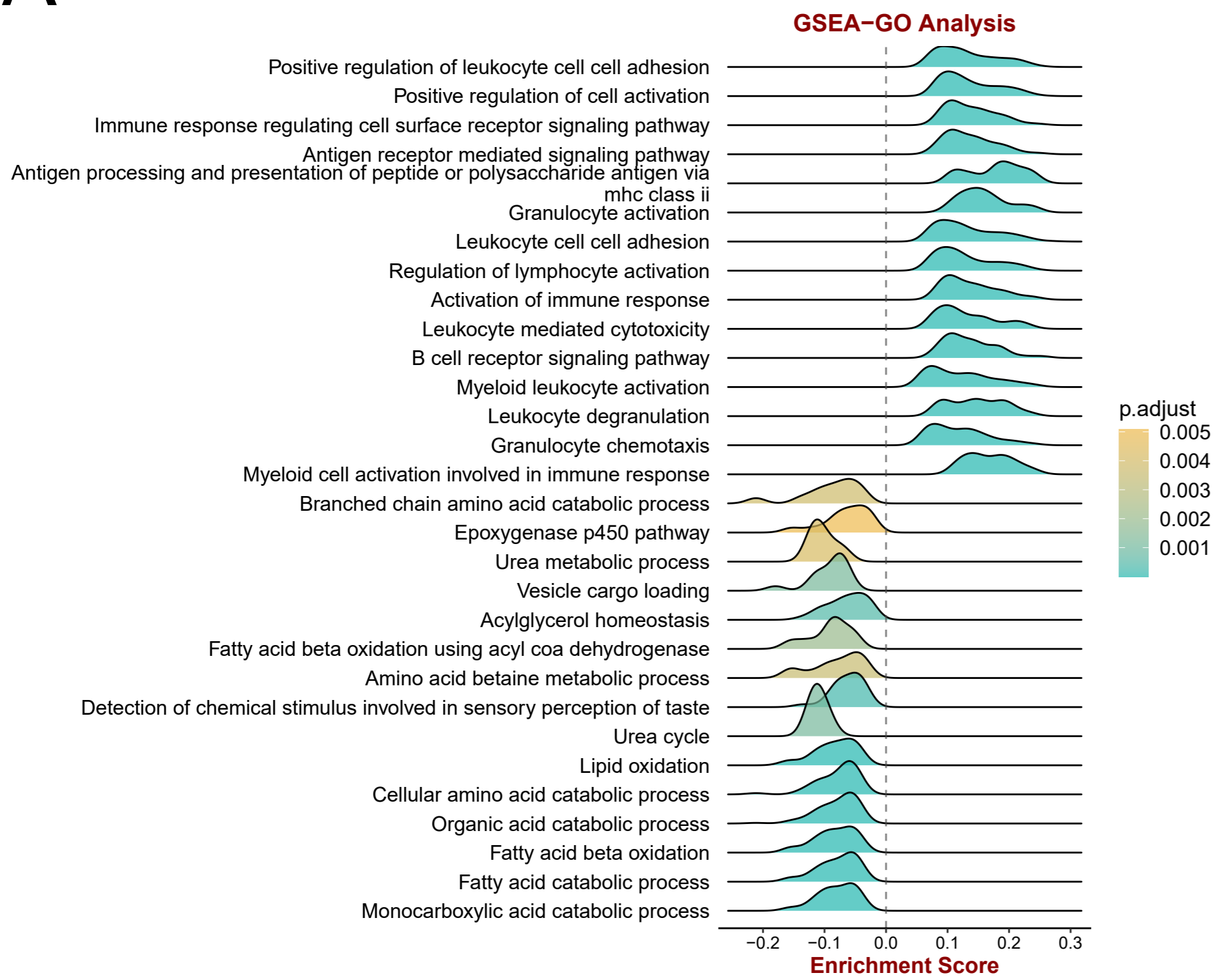

B

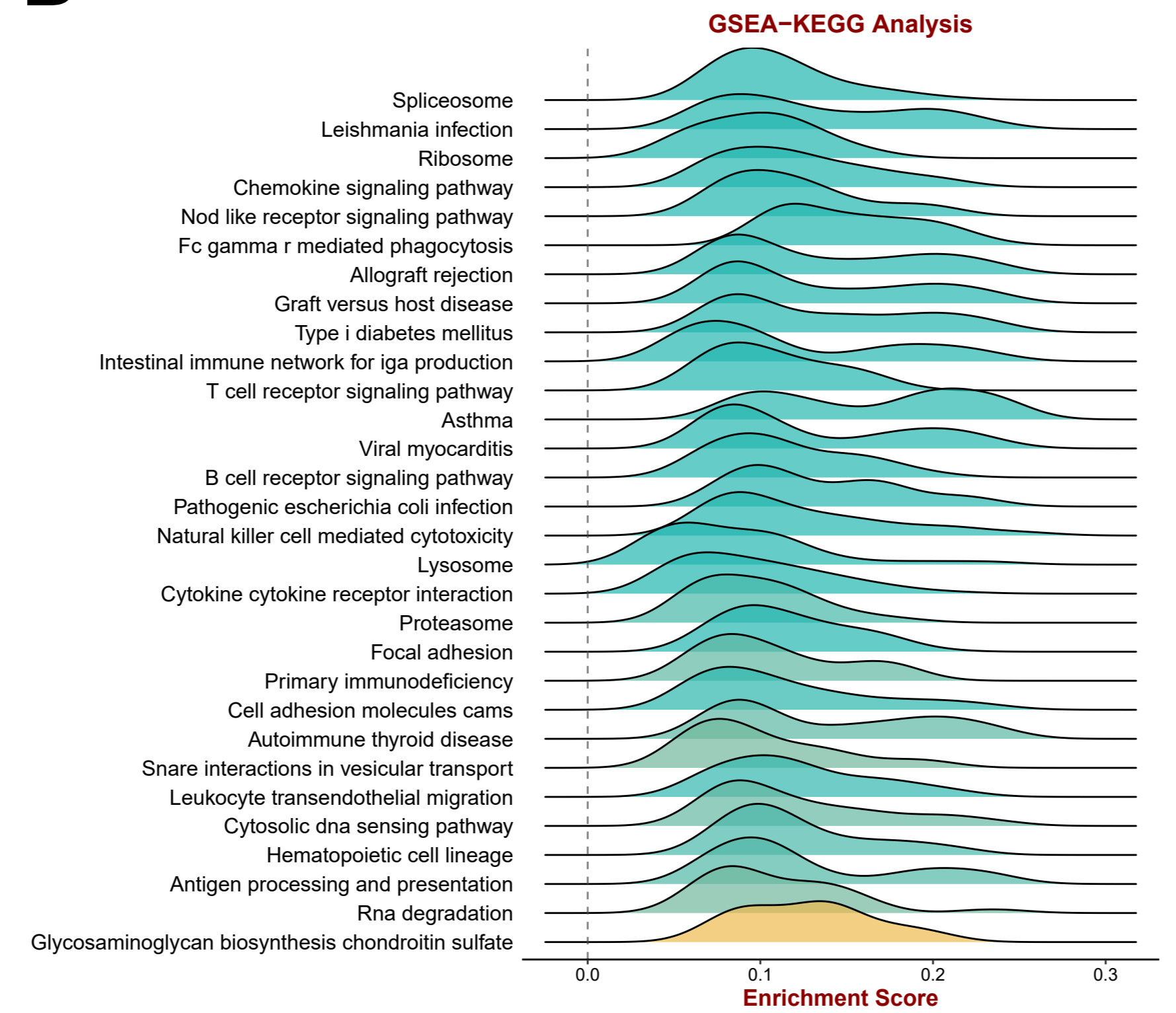

C

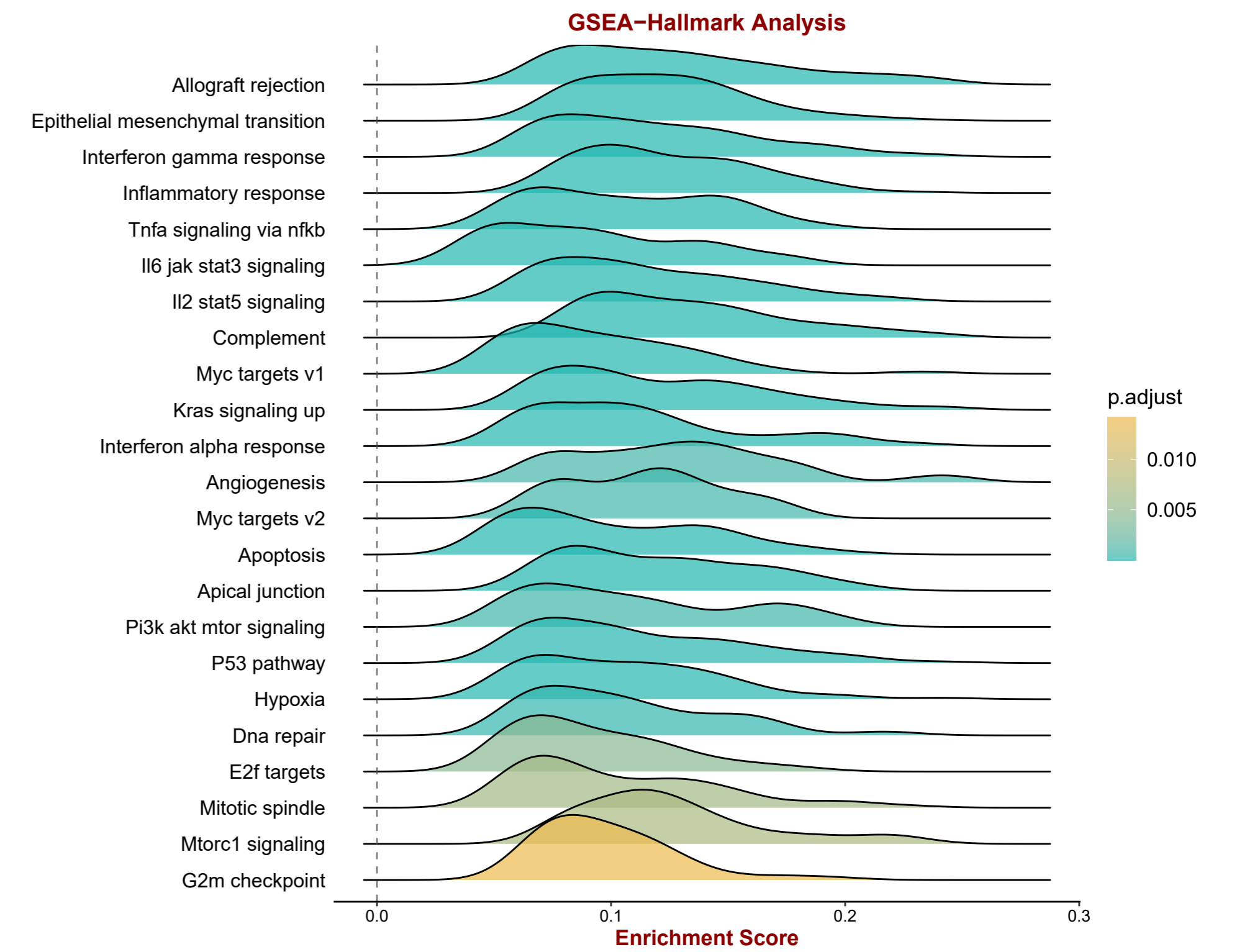

Supplement: Supplementary Figure 2 — (A) The GSEA-GO enrichment profile of CHMP4A in the context of immune regulation, as indicated by the enrichment score. (B) The GSEA-KEGG enrichment profile of CHMP4A in the context of immune regulation, as indicated by the enrichment score; (C) Hallmark gene set enrichment of CHMP4A in LIHC. * P<0.05, ** P<0.01, *** P<0.001, **** P<0.0001. LIHC, Liver Hepatocellular Carcinoma; CHMP4A, Charged Multivesicular Body Protein 4A; ES, Enrichment Score; GSEA, Gene Set Enrichment Analysis; GO, Gene Ontology; KEGG, Kyoto Encyclopedia of Genes and Genomes; CHMP4A, Charged Multivesicular Body Protein 4A; TCGA, The Cancer Genome Atlas. [file Image2.pdf]

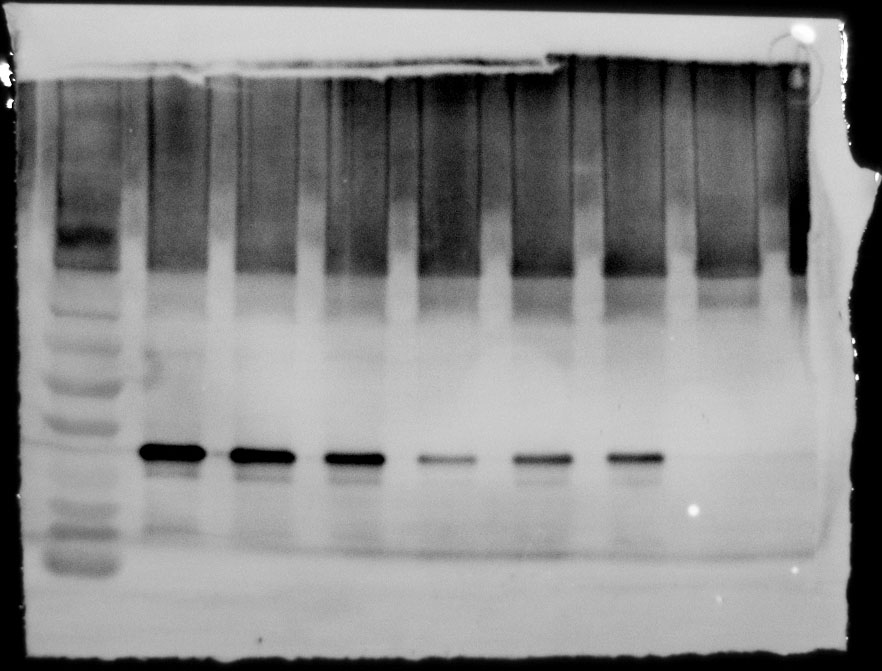

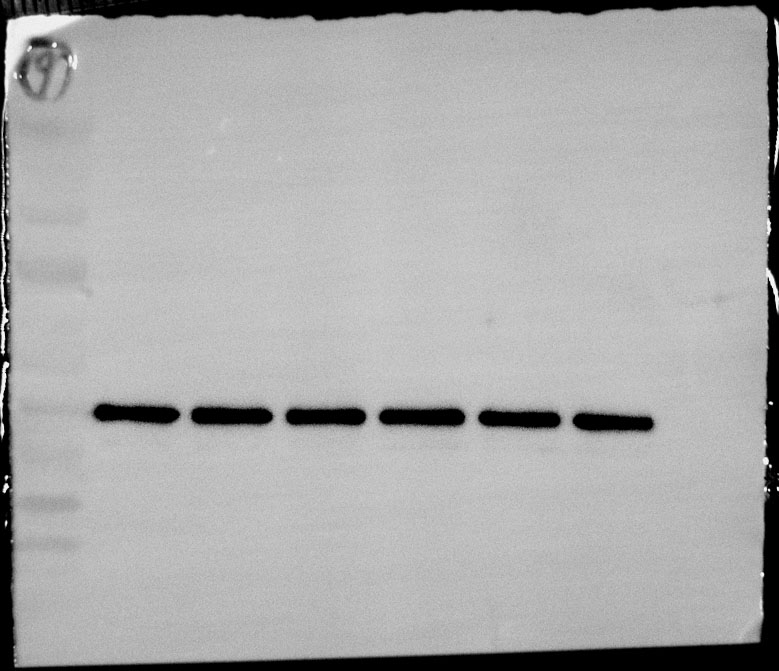

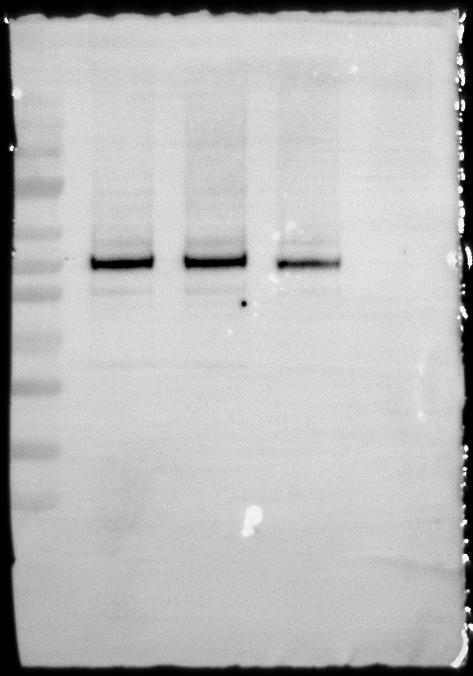

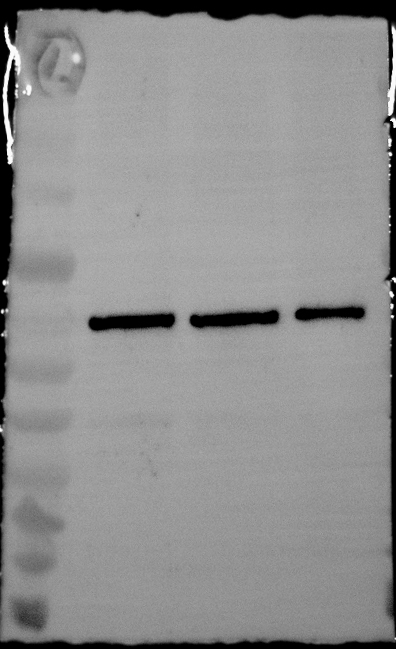

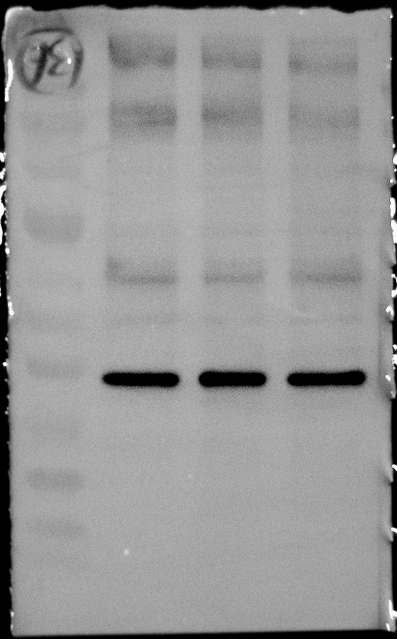

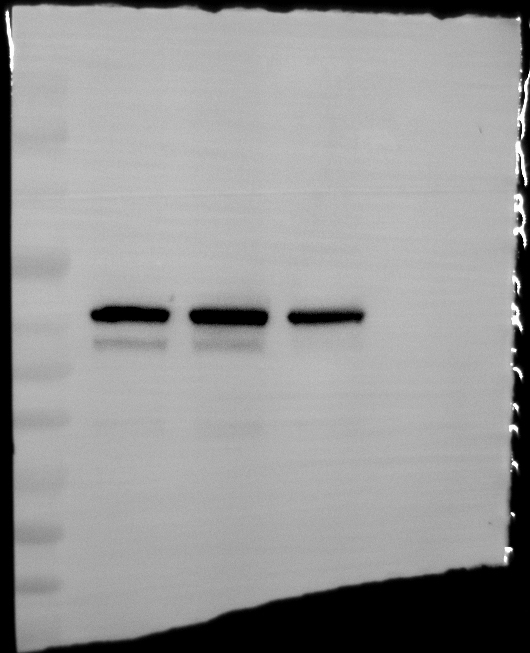

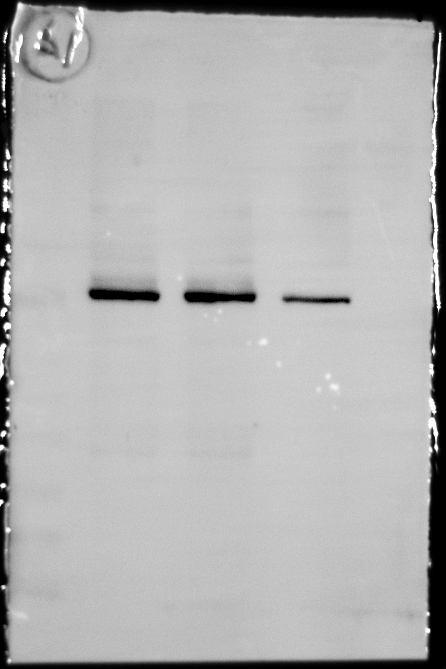

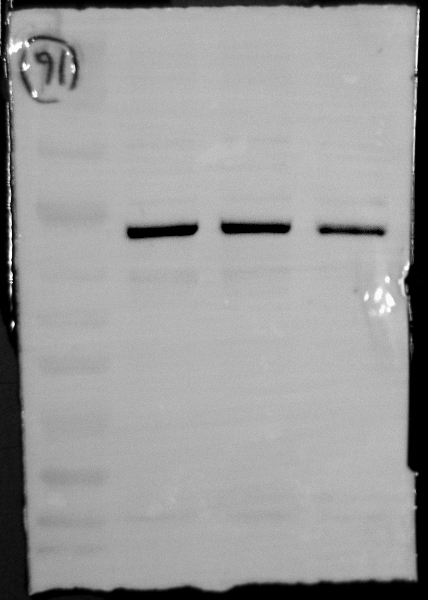

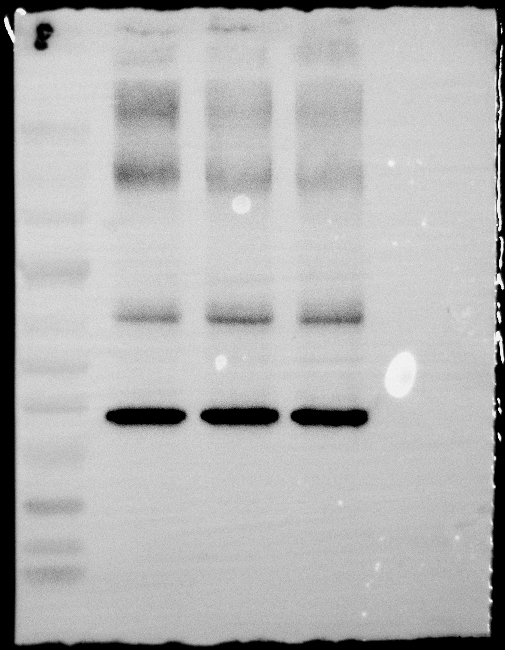

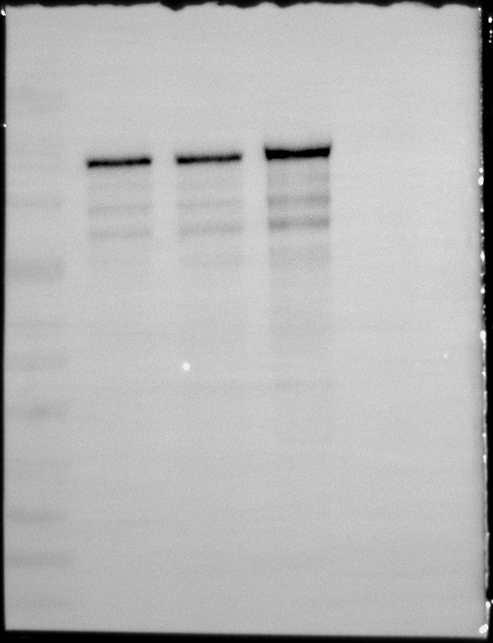

Supplement: Supplementary file 5 [file DataSheet1.docx]
