# Supplementary material for: CHMP4A in hepatocellular carcinoma: exploring its role in tumor progression, immune modulation, and potential link to TIM3 checkpoint
Source: Front Immunol. 2025 Oct 2;16:1682724. doi: 10.3389/fimmu.2025.1682724 (PMC12528155; doi:10.3389/fimmu.2025.1682724)
Supplement: Supplementary file 3 [file Table1.docx]

| Characteristics | overall |
| --- | --- |
| N  Age, n (%) | 474 |
| <= 60 | 177 (47.5%) |
| > 60 | 196 (52.5%) |
| Gender, n (%) |  |
| Female | 121 (32.4%) |
| Male | 253 (67.6%) |
| Pathologic T stage, n (%) |  |
| T1 | 183 (49.3%) |
| T2 | 95 (25.6%) |
| T3 | 80 (21.6%) |
| T4 | 13 (3.5%) |
| Pathologic N stage, n (%) |  |
| N0 | 254 (98.4%) |
| N1 | 4 (1.6%) |
| Pathologic M stage, n (%) |  |
| M0 | 268 (98.5%) |
| M1 | 4 (1.5%) |
| Pathologic stage, n (%) |  |
| Stage I | 173 (49.4%) |
| Stage II | 87 (24.9%) |
| Stage III | 85 (24.3%) |
| Stage IV | 5 (1.4%) |
| AFP(ng/ml), n (%) |  |
| <= 400 | 215 (76.8%) |
| > 400 | 65 (23.2%) |
