# Supplementary material for: CHMP4A in hepatocellular carcinoma: exploring its role in tumor progression, immune modulation, and potential link to TIM3 checkpoint
Source: Front Immunol. 2025 Oct 2;16:1682724. doi: 10.3389/fimmu.2025.1682724 (PMC12528155; doi:10.3389/fimmu.2025.1682724)
Supplement: Supplementary file 4 [file Table2.docx]

**Table S2** **Clinical characteristics of patients with LIHC.**

| Characteristic | No.of patients(%) |
| --- | --- |
| **n** | **16** |
| **Age, n (%)** |  |
| ≤55 | 11(68.8%) |
| >55 | 5 (31.2%) |
| **Gender, n (%)** |  |
| Male | 14(87.5%) |
| Female | 2 (12.5%) |
| **Tumor size, n (%)** |  |
| ≦5cml | 7(43.7%) |
| >5cm | 9(56.3%) |
| **Differentiation, n (%)** |  |
| well | 3(18.7%) |
| Moderate | 9(56.3%) |
| Poor | 4 (25.0%) |
| **Lymph node metastasis, n (%)** |  |
| No | 15 (93.7%) |
| Yes | 1 (6.3%) |
| **TNM stage, n (%)** |  |
| I-II | 14(87.5%) |
| III-IV | 2 (12.5%) |
